# Morning vs Bedtime Dosing and Nocturnal Blood Pressure Reduction in Patients With Hypertension: The OMAN Randomized Clinical Trial

**DOI:** 10.1001/jamanetworkopen.2025.19354

**Published:** 2025-07-09

**Authors:** Runyu Ye, Xiangyu Yang, Xin Zhang, Xianghao Zuo, Yanan Li, Shanshan Jia, Mengzhuo Xu, Lu Liu, Si Wang, Kai Liu, Qingtao Meng, Hang Liao, Zhipeng Zhang, Rufeng Shi, Xinran Li, Xueting Liu, Lirong Sun, Xin Zhang, Qin Ran, Fangfang Chen, Qingyan Gao, Wei Yao, Huamei Shi, Tao Liu, Kun Ma, Li Liu, Kuanqin Chen, Jinquan Gao, Xiaoping Chen

**Affiliations:** 1 Department of Cardiology, West China Hospital, Sichuan University, Chengdu, China; 2 State Key Laboratory of Oral Diseases, National Center for Stomatology, National Clinical Research Center for Oral Diseases, West China Hospital of Stomatology, Sichuan University, Chengdu, Sichuan, China; 3 Department of Anesthesiology, West China Hospital, Sichuan University, Chengdu, China; 4 Department of Cardiology, Chengdu Seventh People’s Hospital, Chengdu, China; 5 Medical Treatment Alliance of West China Hospital, Sichuan University and Qinglong Community Healthcare Center, Chengdu, China; 6 Department of Cardiology, Medical Treatment Alliance of West China Hospital, Sichuan University and Jiulong County People’s Hospital, Jiulong, China; 7 Department of Cardiology, Medical Treatment Alliance of West China Hospital, Sichuan University and Luhuo County People’s Hospital, Luhuo, China; 8 Department of Cardiology, Nanchong Central Hospital and Affiliation of Beijing Anzhen Hospital, Capital Medical University, Nanchong, China; 9 Medical Treatment Alliance of West China Hospital, Sichuan University and Shuangqiaozi Community Healthcare Center, Chengdu, China; 10 Department of Cardiology, The Second People’s Hospital of Yibin and Yibin Affiliated Hospital of West China Hospital, Sichuan University, Chengdu, China; 11 Department of Cardiology, Medical Treatment Alliance of West China Hospital, Sichuan University and Litang County People’s Hospital, Litang, China; 12 Department of Cardiology, Chongzhou People’s Hospital, Chengdu, China

## Abstract

**Importance:**

Nocturnal blood pressure is difficult to manage in clinical practice. Antihypertensive chronotherapy may offer a potential approach for better control. However, the clinical evidence supporting this approach remains controversial.

**Objective:**

To compare the effects of morning vs bedtime antihypertensive medication administration on nocturnal blood pressure reduction and circadian rhythm among patients with hypertension.

**Design, Setting, and Participants:**

This randomized clinical trial was conducted at 15 hospitals in China from June 1, 2022, to April 30, 2024, with a 12-week follow-up. Patients with hypertension without prior antihypertensive treatment or who had discontinued antihypertensive agents for 2 weeks were randomized to the morning (6:00-10:00 am) or bedtime (6:00-10:00 pm) dosing group.

**Interventions:**

Patients received a single pill containing olmesartan, 20 mg, and amlodipine, 5 mg, daily for 12 weeks, with dosage adjustments based on ambulatory and office blood pressure measurements at week 4 and week 8.

**Main Outcomes and Measures:**

The primary outcome was the change in nighttime systolic blood pressure from baseline to 12 weeks. Key secondary outcomes included changes in office and other ambulatory blood pressure indicators. The primary and secondary outcomes were analyzed in the intention-to-treat and the per-protocol populations.

**Results:**

A total of 720 patients (mean [SD] age, 55.5 [10.6] years; 409 men [56.8%]) were randomized to morning (n = 352) or bedtime (n = 368) dosing groups. The mean (SD) baseline blood pressure values for morning vs bedtime dosing at 24 hours were 148.0 (11.1)/91.4 (9.0) mm Hg vs 147.6 (11.0)/91.6 (9.2) mm Hg, for daytime were 152.3 (11.0)/94.0 (9.2) mm Hg vs 151.5 (11.6)/94.0 (9.8) mm Hg, for nighttime were 138.4 (15.1)/85.4 (10.4) mm Hg vs 138.3 (13.0)/85.8 (9.4) mm Hg, and in the office were 154.4 (12.1)/94.6 (10.3) mm Hg vs 154.3 (12.5)/95.1 (11.1) mm Hg. Compared with patients in the morning dosing group, those in the bedtime dosing group showed significantly greater reductions in nighttime systolic blood pressure (between-group difference, −3.0 mm Hg [95% CI, −5.1 to −1.0 mm Hg]; *P* = .004), and nighttime diastolic blood pressure (between-group difference, −1.4 mm Hg [95% CI, −2.8 to −0.1 mm Hg]; *P* = .04), with better nocturnal systolic blood pressure control (79.0% [244 of 309] vs 69.8% [208 of 298]; *P* = .01) and improved circadian rhythm. The incidence of nocturnal hypotension did not differ.

**Conclusions and Relevance:**

In this randomized clinical trial of antihypertensive chronotherapy, bedtime dosing provided better control of nocturnal blood pressure and improved the circadian rhythm, without reducing the efficacy on mean daytime or 24-hour blood pressure, or increasing the risk of nocturnal hypotension. These findings support the potential advantages of bedtime administration and offer new evidence to guide future research on antihypertensive chronotherapy.

**Trial Registration:**

Chinese Clinical Trial Registry Identifier: ChiCTR2200059719

## Key Points

**Question:**

Can bedtime dosing of antihypertensive drugs in patients with hypertension provide better control of nocturnal blood pressure and improve the circadian rhythm compared with morning dosing?

**Findings:**

In this multicenter randomized clinical trial comprising 720 patients with hypertension, those in the bedtime dosing group achieved better blood pressure control and circadian rhythm than the morning dosing group.

**Meaning:**

Findings of this study provide valuable insights into the application of antihypertensive chronotherapy for the population with hypertension, especially in the context of enhancing the management of nocturnal blood pressure.

## Teaser

This randomized clinical trial compares the effect of morning vs bedtime administration of antihypertensive agents on nocturnal blood pressure control and circadian rhythm among patients with hypertension.

## Introduction

Hypertension is a leading risk factor for cardiovascular diseases.^1^ There are approximately 300 million patients in China with hypertension, with a control rate of only 16.8%.^2^ Currently, single-pill combination antihypertensive agents are widely recommended by guidelines for hypertension management as the initial treatment choice to improve treatment adherence and increase the control rate.^3,4^ Olmesartan-amlodipine is a novel single-pill combination of an angiotensin receptor blocker and a calcium-channel blocker with a long-term and stable antihypertensive effect. Previous research has confirmed that olmesartan-amlodipine can significantly reduce blood pressure (BP) among patients with hypertension who fail to meet BP targets with monotherapy.^5^

Nighttime BP assessed by ambulatory BP monitoring (ABPM) is a more sensitive prognostic indicator of cardiovascular events than office, 24-hour, or daytime BP.^6^ Moreover, it has been recognized that a diminished nocturnal BP decrease less than 10% of daytime mean BP can translate into a greater risk of organ damage and cardiovascular events.^7,8^ However, due to the difficulty of nocturnal BP management, uncontrolled nocturnal BP is particularly common among treated patients with hypertension, with an incidence rate between 30% and 60%.^9,10^ Achieving nocturnal BP control has significant potential in reducing the risk of cardiovascular events and mortality among patients with hypertension. Some guidelines have recommended nocturnal BP control as an important strategy for high-quality hypertension management.^11^

Considering the optimal timing for administering antihypertensive agents, antihypertensive chronotherapy may provide an approach for effectively controlling nocturnal BP. Studies have shown that evening dosing of antihypertensive drugs can be more effective than morning dosing in lowering 24-hour BP, normalizing the diurnal rhythm, and reducing the risk of cardiovascular mortality.^12,13^ However, other research indicates that the timing of administration of antihypertensive medication does not significantly affect BP levels, nor does it influence major cardiovascular outcomes.^14,15,16^ The evidence for antihypertensive chronotherapy now shows high heterogeneity, including differences in the population, intervention methods, and evaluation indicators.^15,17,18^ Thus, the clinical benefit of bedtime dosing of antihypertensive drugs remains controversial.

In the The Effects of Olmesartan/Amlodipine Administered in the Morning or at Night on Nocturnal Blood Pressure Reduction in Chinese Patients With Mild-Moderate Essential Hypertension (OMAN) Trial, we compared the effects of morning vs bedtime administration of the olmesartan-amlodipine tablet on nocturnal BP reduction and recovery of circadian rhythm among patients with hypertension, aiming to provide further evidence for antihypertensive chronotherapy and enhance nocturnal BP management.

## Methods

### Study Design and Participants

This prospective, multicenter, randomized, open-label, parallel-group, superiority clinical trial was conducted at 15 hospitals in China between June 1, 2022, and April 30, 2024. Patients with hypertension aged 18 to 75 years without prior antihypertensive treatment or who had discontinued antihypertensive agents for 2 weeks were eligible. The study protocol is described in Supplement 1. The exclusion criteria are detailed in eTable 1 in Supplement 2. The study was approved by the Biomedical Research Ethics Committee of the West China Hospital of Sichuan University, and registered at Chinese Clinical Trial Registry (ChiCTR2200059719). All participants provided written informed consent. The study followed the Consolidated Standards of Reporting Trials (CONSORT) reporting guideline.

### Randomization

Randomization was performed by specialized staff from the main research unit using the Research Randomizer,^19^ which generated random numbers for each research unit. Participants from each unit were then randomly assigned in a 1:1 ratio to the morning or the bedtime administration group.

### Sample Size Calculation

The sample size was calculated based on the primary end point, which was the difference in the change in nighttime SBP between the 2 groups from baseline to 12 weeks. The study was powered to detect a difference of 3 mm Hg in nighttime SBP.^20^ We assumed an SD of 12 mm Hg, set the significance level at *P* < .05 (2-sided), and aimed for a power of 85%. Consequently, 289 patients were needed in each group. Assuming a 20% dropout rate, the total sample size was adjusted to 720 patients.

### Procedures

**Figure 1.**
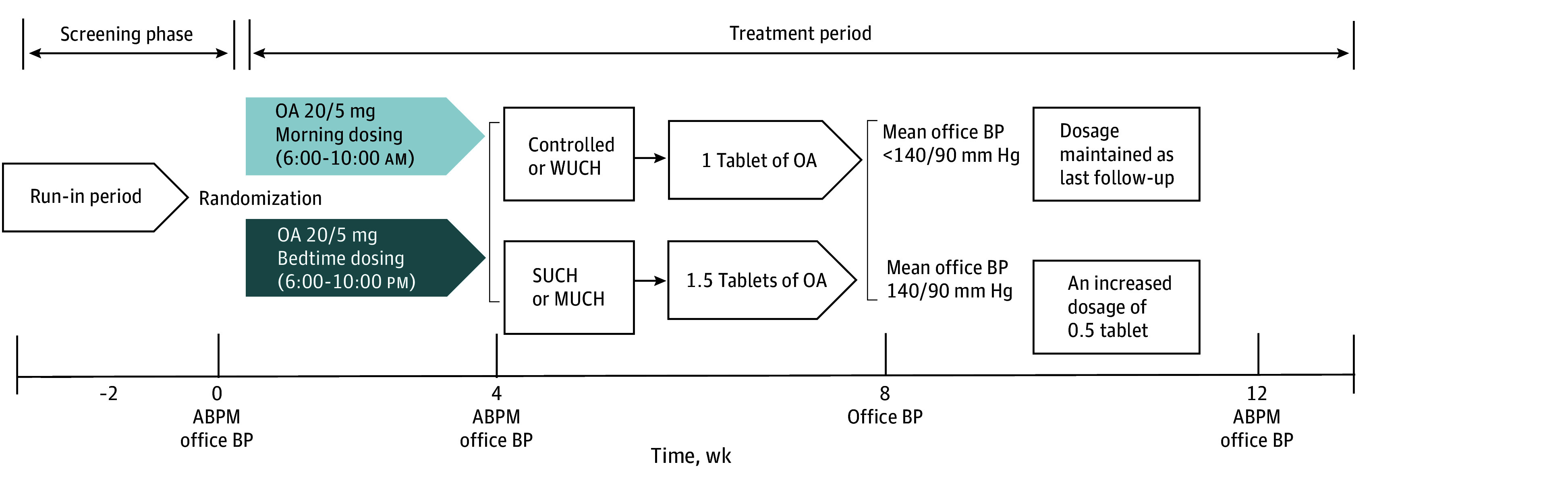
Study Design ABPM indicates ambulatory blood pressure monitoring; BP, blood pressure; MUCH, masked uncontrolled hypertension; OA 20/5 mg, combination tablet with 20 mg of olmesartan and 5 mg of amlodipine; SUCH, sustained uncontrolled hypertension; and WUCH, white coat uncontrolled hypertension (blood pressure is elevated when measured in a medical setting, but normal when measured outside of that setting, such as at home).

Eligible participants were randomized in a 1:1 ratio to either a morning (6-10 am) or a bedtime (6-10 pm) administration group. Each participant received 1 tablet of olmesartan-amlodipine (olmesartan, 20 mg, and amlodipine, 5 mg) daily as the initial treatment for 4 weeks and was then followed up every 4 weeks for 12 weeks. During follow-up, the olmesartan-amlodipine dosage was adjusted based on the ABPM and office BP monitoring (OBPM) values (Figure 1). Controlled BP was defined as 24-hour BP less than 130/80 mm Hg, daytime BP less than 135/85 mm Hg, nighttime BP less than 120/70 mm Hg, and OBPM less than 140/90 mm Hg.^3^ At the first follow-up visit (week 4), the olmesartan-amlodipine dosage was adjusted to 1.5 tablets per day for patients with masked uncontrolled hypertension or sustained uncontrolled hypertension. Masked uncontrolled hypertension was defined as normal office BP but elevated BP on ABPM readings. Sustained uncontrolled hypertension was defined as elevated BP on both office BP and ABPM readings. If office BP remained uncontrolled after an additional 4 weeks (week 8), the olmesartan-amlodipine dosage was further increased by 0.5 tablet per day. Participant-reported adherence to randomized dosing time was recorded during each follow-up visit.

### ABPM and Other Assessments

Baseline data on demographic characteristics and follow-up data were uploaded to an internet-based hypertension management system (Red Shine Chronic Disease Management System; Hypertension Center of West China Hospital).^20^ Office BP was measured with a calibrated electronic sphygmomanometer (HBP-1100-E; Omron Corp) according to the guidelines.^3,4^ Blood pressure was measured 3 times and recorded in the electronic system, with the mean of the last 2 values automatically calculated as mean office BP. Ambulatory BP monitoring was performed at baseline, week 4, and week 12 using a validated device (TM2430; A&D Inc) following procedures recommended by the guidelines.^21,22^ Blood pressure was measured at 20-minute intervals between 6 am and 10 pm, and at 30-minute intervals between 10 pm and 6 am. Participants were instructed to record their daily schedule on a diary card, including the times they went to bed and woke up. The definitions of ABPM-related indexes are in Supplement 1.

### Outcomes

The primary outcome was the difference in the change in the mean nighttime SBP from baseline to 12 weeks. Secondary outcomes included reduction in nighttime diastolic BP (DBP); reductions in 24-hour, daytime, morning, and office SBP and DBP; control rates of ABPM and OBPM; proportion of patients with a nocturnal BP decrease less than 10% of daytime mean BP; BP load reduction; treatment response rate (defined as SBP<140 mm Hg or a decrease of ≥20 mm Hg, and DBP<90 mm Hg or a decrease of ≥10 mm Hg);^23^ and proportion of patients requiring intensive treatment.

### Statistical Analysis

Quantitative variables were presented as the mean (SD) values or as the median (IQR) values based on the data distribution. Qualitative variables were presented as numbers and percentages. Quantitative variables were compared between the 2 groups using the *t* test or the Mann-Whitney test, while the χ^2^ test or the Fisher exact test was used for qualitative variables.

The primary and secondary outcomes were analyzed in the intention-to-treat population. Missing office and ABPM values were imputed using the last observation carried forward method. In addition, sensitivity analyses using multiple imputation were conducted for patients with missing data, with the values imputed by a regression model or chained equations using corresponding baseline BP, treatment group, age, sex, and body mass index (BMI) as covariates. A preliminary analysis of colinearity was performed among covariates. Primary and secondary outcomes were also assessed in the per-protocol population.

The primary and secondary outcomes were analyzed using an analysis of covariance model adjusted for corresponding baseline BP. A linear mixed-effects model was also used, adjusting for corresponding baseline BP and study site, with site as a random effect, to evaluate the treatment effects considering possible site-level heterogeneity. Subgroup analysis included age (65 years as threshold), sex, baseline office BP (above or below mean SBP), BMI, comorbidities, drinking status, and smoking status. Statistical analyses were performed using SPSS, version 26.0 (IBM Corp) and R, version 4.1.0 (R Project for Statistical Computing). All *P* values were from 2-sided tests and *P* < .05 was considered statistically significant.

## Results

### Baseline Characteristics of Study Participants

**Figure 2.**
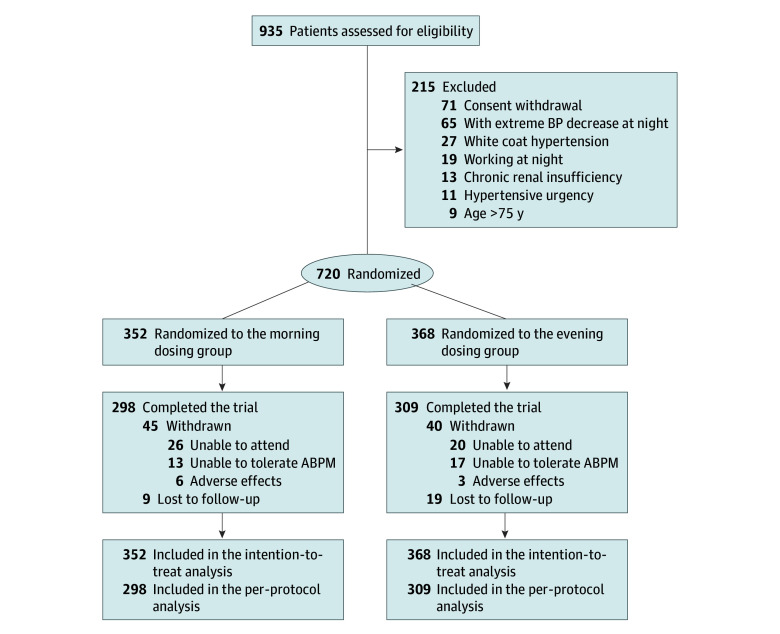
Flow Diagram of the OMAN (The Effects of Olmesartan/Amlodipine Administered in the Morning or at Night on Nocturnal Blood Pressure Reduction in Chinese Patients With Mild-Moderate Essential Hypertension) Trial White coat hypertension is when blood pressure (BP) is elevated when measured in a medical setting, but normal when measured outside of that setting, such as at home. ABPM indicates ambulatory blood pressure monitoring.

We included 720 participants in the study (mean [SD] age, 55.5 [10.6] years; 409 men [56.8%] and 311 women [43.2%]) (Table). Baseline characteristics were balanced between groups. A total of 546 patients (75.8%) were younger than 65 years. The mean (SD) baseline BP values for morning vs bedtime dosing at 24 hours were 148.0 (11.1)/91.4 (9.0) mm Hg vs 147.6 (11.0)/91.6 (9.2) mm Hg, for daytime were 152.3 (11.0)/94.0 (9.2) mm Hg vs 151.5 (11.6)/94.0 (9.8) mm Hg, for nighttime were 138.4 (15.1)/85.4 (10.4) mm Hg vs 138.3 (13.0)/85.8 (9.4) mm Hg, and in the office were 154.4 (12.1)/94.6 (10.3) mm Hg vs 154.3 (12.5)/95.1 (11.1) mm Hg. From June 2022 to January 2024, 935 patients were screened, with 720 enrolled and randomly assigned to the morning (n = 352) or bedtime (n = 368) dosing groups (Figure 2). The post hoc power analysis indicated that the study achieved a power (1 − β) of 98%. Of the 720 patients randomized, 607 (84.3%) completed the 12-week follow-up. Comparison of participants at follow-up and those lost to follow-up is in eTable 2 in Supplement 2.

**Table.** Baseline Characteristics of Study Participants

Characteristic	All patients (N = 720)	Dosing	
		Morning (n = 352)	Bedtime (n = 368)
Age, mean (SD), y	55.5 (10.6)	55.6 (11.0)	55.4 (10.3)
Sex, No. (%)			
Male	409 (56.8)	206 (58.5)	203 (55.2)
Female	311 (43.2)	146 (41.5)	165 (44.8)
BMI, mean (SD)	26.0 (3.4)	26.1 (3.4)	26.0 (3.5)
Total cholesterol, mean (SD), mg/dL	196.1 (42.6)	194.4 (41.0)	197.8 (44.2)
LDL cholesterol, mean (SD), mg/dL	114.7 (35.1)	115.4 (35.6)	114.0 (34.7)
HDL cholesterol, mean (SD), mg/dL	50.9 (15.9)	51.8 (16.8)	50.1 (14.9)
Triglycerides, median (IQR), mg/dL	143.4 (102.9-204.5)	143.9 (102.7-202.8)	142.1 (108.0-208.5)
Plasma glucose, median (IQR), mg/dL	96.8 (88.5-108.4)	96.8 (88.1-108.3)	97.1 (89.4-109.2)
Current smoker, No. (%)	155 (21.5)	85 (24.1)	70 (19.0)
Current drinker, No. (%)	189 (26.3)	99 (28.1)	90 (24.5)
Diabetes, No. (%)	121 (16.8)	55 (15.6)	66 (17.9)
Duration of hypertension, median (IQR), y	2.0 (0.0-6.0)	3.0 (0.0-6.0)	2.0 (0.0-6.0)
History of antihypertensive medications use	395 (54.9)	189 (53.7)	206 (56.0)
Hypertension treatment, No. (%)			
ACEI	17 (4.3)	8 (4.2)	9 (4.4)
ARB	226 (57.2)	112 (59.3)	114 (55.3)
β-Blocker	40 (10.1)	18 (9.5)	22 (10.7)
CCB	258 (65.3)	120 (63.5)	138 (67.0)
Diuretic	29 (7.3)	14 (7.4)	15 (7.3)
Baseline BP, mean (SD), mm Hg			
Office			
SBP	154.3 (12.4)	154.4 (12.2)	154.3 (12.5)
DBP	94.9 (10.7)	94.6 (10.3)	95.1 (11.1)
PP	78.3 (11.1)	78.3 (11.0)	78.3 (11.2)
24-h			
SBP	147.8 (11.0)	148.0 (11.1)	147.6 (11.0)
DBP	91.5 (9.1)	91.4 (9.0)	91.6 (9.2)
PP	76.0 (9.7)	76.0 (9.8)	76.1 (9.7)
Daytime			
SBP	151.9 (11.3)	152.3 (11.0)	151.5 (11.6)
DBP	94.0 (9.5)	94.0 (9.2)	94.0 (9.8)
PP	79.4 (10.2)	79.3 (10.1)	79.5 (10.2)
Nighttime			
SBP	138.4 (14.1)	138.4 (15.1)	138.3 (13.0)
DBP	85.6 (9.9)	85.4 (10.4)	85.8 (9.4)
PP	68.0 (9.9)	68.2 (10.0)	67.8 (9.8)
Morning			
SBP	149.8 (15.2)	150.0 (15.2)	149.5 (15.2)
DBP	93.5 (11.6)	93.0 (11.6)	93.9 (11.5)
PP	75.5 (11.6)	75.1 (11.7)	75.8 (11.6)
No nocturnal SBP decrease <10% of daytime mean BP or with increase in nocturnal SBP, No. (%)	375 (52.1)	179 (50.9)	196 (53.3)
No nocturnal DBP decrease <10% of daytime mean BP or with increase in nocturnal DBP, No. (%)	395 (54.9)	192 (54.5)	203 (55.2)
24-h BP load, mean (SD), %			
SBP	81.3 (15.7)	81.3 (15.5)	81.3 (15.8)
DBP	77.8 (19.5)	77.7 (19.4)	77.9 (19.7)
Daytime BP load, mean (SD), %			
SBP	81.3 (17.3)	82.1 (15.8)	80.6 (18.6)
DBP	74.0 (22.7)	74.3 (22.4)	73.7 (23.1)
Nighttime BP load, mean (SD), %			
SBP	82.8 (20.4)	81.4 (22.8)	84.0 (17.7)
DBP	88.0 (17.7)	86.9 (18.9)	89.0 (16.3)
Nocturnal BP decrease, median (IQR), mm Hg			
SBP	9.7 (4.1-13.9)	9.8 (4.1-14.6)	9.5 (4.1-13.5)
DBP	8.8 (3.8-13.7)	8.72 (3.6-14.4)	8.9 (4.1-13.3)

Abbreviations: ACEI, angiotensin converting enzyme inhibitor; ARB, angiotensin receptor blocker; BMI, body mass index (calculated as weight in kilograms divided by height in meters squared); BP, blood pressure; CCB, calcium-channel blocker; DBP, diastolic blood pressure; HDL, high-density lipoprotein; LDL, low-density lipoprotein; PP, pulse pressure; SBP, systolic blood pressure.

SI conversion factors: To convert total cholesterol, LDL cholesterol, and HDL cholesterol to millimoles per liter, multiply by 0.0259; triglycerides to millimoles per liter, multiply by 0.0113; and glucose to millimoles per liter, multiply by 0.0555.

### Primary Outcomes

**Figure 3.**
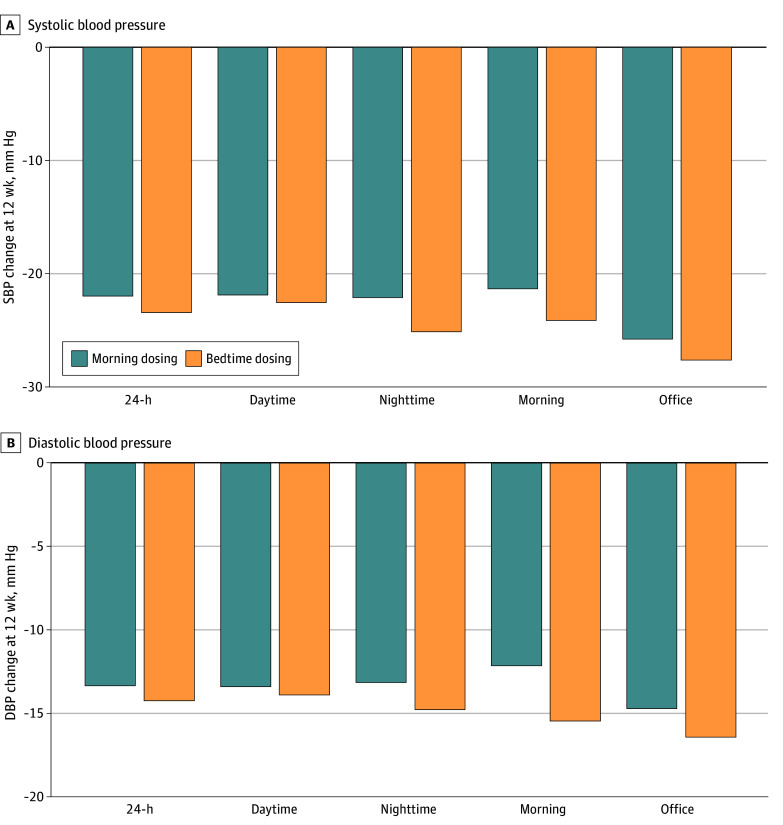
Changes in Ambulatory and Office Blood Pressure Values From Baseline to 12 Weeks DBP indicates diastolic blood pressure; SBP, systolic blood pressure.

The mean (SD) change from baseline to 12 weeks in nighttime SBP was significantly greater in the bedtime dosing group (−25.3 [15.0] mm Hg vs −22.3 [16.8] mm Hg; between-group difference, −3.0 mm Hg [95% CI, −5.1 to −1.0 mm Hg]; *P* = .004) (Figure 3; eTable 3 in Supplement 2). These results were consistent in the per-protocol population (between-group difference, −3.4 mm Hg [95% CI, −5.3 to −1.5 mm Hg]; *P* = .001) (eTable 4 in Supplement 2) and in the sensitivity analyses (eTables 5 and 6 in Supplement 2).

### Secondary Outcomes

The mean BP values for each group during the study are shown in eFigures 1 and 2 in Supplement 2. At week 12, patients in the bedtime dosing group exhibited significantly greater reductions from baseline in nighttime DBP (−1.4 mm Hg [95% CI, −2.8 to −0.1 mm Hg]), morning SBP (−3.1 mm Hg [95% CI, −5.6 to −0.5 mm Hg]), and morning DBP (−2.7 mm Hg [95% CI, −4.4 to −1.0 mm Hg]) (Figure 3). These findings were consistent in the per-protocol population (eTable 4 in Supplement 2).

**Figure 4.**
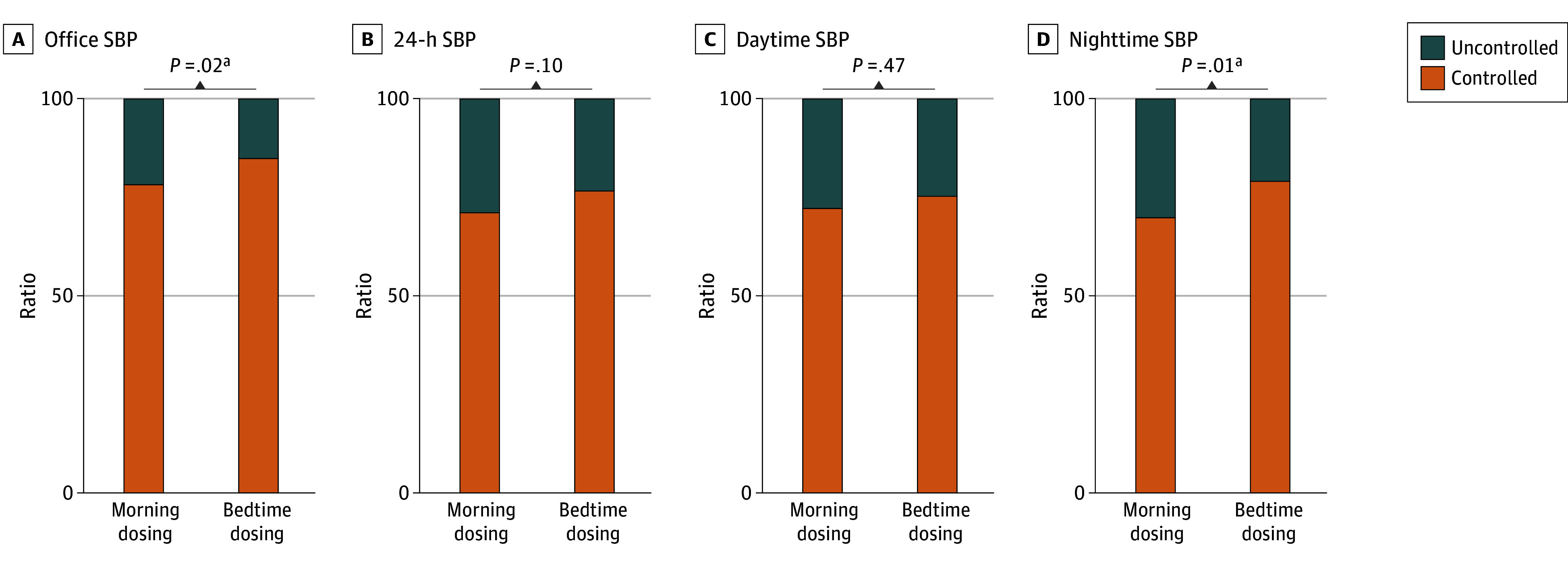
Control Rates of Ambulatory and Office Blood Pressure at Week 12 SBP indicates systolic blood pressure. ^a^Statistically significant.

At week 12, patients in the bedtime dosing group also demonstrated a better nocturnal SBP control rate (79.0% [244 of 309] vs 69.8% [208 of 298]; *P* = .01), and office SBP control rate (88.7% [274 of 309] vs 82.2% [245 of 298]; *P* = .02) compared with the morning dosing group (Figure 4; eFigure 3 in Supplement 2). In addition, patients in the bedtime dosing group showed a greater decrease in nighttime BP load (eTable 8 in Supplement 2). The proportion of patients without a nocturnal BP decrease less than 10% of the daytime mean BP or with increased nocturnal SBP decreased from 53.3% (196 of 368) to 36.9% (114 of 309) in the bedtime dosing group (*P* < .001), while there was no significant change in the morning dosing group (decreased from 50.9% [179 of 352] to 45.3% [135 of 298]; *P* = .35) (eFigure 4 in Supplement 2). The 24-hour BP patterns at baseline and after 12 weeks of intervention for both groups are shown in eFigure 5 in Supplement 2. Nighttime SBP and morning SBP in the bedtime dosing group were lower compared with the morning dosing group, indicating a significant improvement in BP patterns.

Regarding treatment response rate, a higher proportion of patients in the bedtime dosing group responded favorably to OBPM at week 12, while no differences were observed at week 4 or week 8 (eTable 9 in Supplement 2). During the final follow-up, the mean (SD) number of tablets was higher in the morning dosing group compared with the bedtime dosing group (1.5 [0.4] vs 1.4 [0.4]; *P* = .02). At week 4, the dosage for 56.9% of participants (391 of 687) was titrated to 1.5 tablets (morning vs bedtime: 61.5% [203 of 330] vs 52.7% [188 of 357]; *P* = .02). At week 8, the dosage for 28.4% of participants (183 of 644) was titrated to 2 tablets (32.1% [101 of 315] vs 24.9% [82 of 329]; *P* = .04) (eTable 10 in Supplement 2).

Subgroup analysis suggested that the between-group difference was significantly greater for bedtime dosing among male patients, those older than 65 years, nondrinkers, nonsmokers, those with a BMI of 24 or more(calculated as weight in kilograms divided by height in meters squared), and those with a baseline office SBP of 155 mm Hg or more (eFigure 6 in Supplement 2).

### Treatment Safety and Compliance

No significant differences were observed in the prevalence of reported adverse effects and nocturnal hypotension between the groups during follow-up (eTable 9-11 in Supplement 2). Poor adherence to the assigned dosing time was reported by 4.5% (16 of 352) of patients in the morning dosing group and 5.4% (20 of 368) in the bedtime dosing group (*P* = .58). Patients who forgot to take their pills were instructed to resume the prescribed dosage and complete the follow-up.

## Discussion

The OMAN trial was a multicenter randomized clinical trial investigating the effects of antihypertensive chronotherapy using a long-acting single-pill combination as the initial treatment regimen. Our study found that bedtime dosing can better control of nocturnal BP and improve the circadian rhythm compared with morning dosing, without compromising the efficacy on mean daytime or 24-hour BP or increasing the risk of nocturnal hypotension.

Antihypertensive chronotherapy is a prominent topic in the field of hypertension worldwide. However, various studies on this topic have yielded differing results.^12,13,15,16^ A recently published meta-analysis found that evening dosing of antihypertensive drugs was significantly associated with reduced ambulatory BP values and cardiovascular events.^13^ Most of the original research included in the meta-analysis was conducted by Hermida et al.^12^ The Hygia Chronotherapy Trial, the largest study by Hermida et al^12^ so far exploring the effects of chronotherapy on ABPM-related indicators, reported that taking at least 1 BP-lowering drug at bedtime can result in better ABPM control and reduce cardiovascular disease risk compared with taking all drugs on waking. However, this study raised concerns about its methods and plausibility of the effect sizes.^24,25^ Conversely, the Treatment In Morning vs Evening (TIME) study found no significant difference in major cardiovascular outcomes between evening and morning dosing of antihypertensive agents.^16^ However, the TIME study had some limitations, including enrolling patients already taking antihypertensive medications and not performing guideline-recommended ABPM at baseline to confirm hypertension diagnosis, warranting further investigation. In our study, 24-hour ABPM was conducted both at baseline and after treatment, providing robust data on the differences in nocturnal BP reduction between morning and bedtime dosing. In addition, the 3.0-mm Hg difference in nighttime SBP reductions observed in our study is consistent with the findings of the previous meta-analysis, which reported a difference of 3.5 mm Hg (95% CI, 1.3-5.6 mm Hg) after excluding data from Hermida et al.^13^ Considering that a reduction of 2 to 5 mm Hg in office SBP is associated with a 7% to 10% reduction in the risk of cardiovascular events,^26,27^ we hypothesize that the 3-mm Hg difference in nighttime SBP observed between groups may potentially lower long-term cardiovascular risk. However, further studies with larger sample sizes are warranted to verify the effects of nighttime BP reduction on cardiovascular outcomes.

The results may be related to the following potential mechanisms. First, due to the circadian BP rhythm, a decrease in nocturnal BP may lead to a decrease in renal perfusion and activate the renin-angiotensin-aldosterone system. Therefore, taking olmesartan at bedtime may better inhibit nocturnal activation of the renin-angiotensin-aldosterone system.^28,29^ Second, amlodipine, when taken at bedtime, takes advantage of its pharmacokinetic characteristics, reaching peak blood drug concentration in 6 to 12 hours, enhancing its antihypertensive effect at night. Amlodipine also has a longer half-life and bioavailability when administered at bedtime than during the day, indicating increased absorption.^17,30^ Thus, the combination of olmesartan and amlodipine could better control nocturnal and morning BP and improve the circadian rhythm of BP, without diminishing the efficacy on mean daytime or 24-hour SBP. In our study, patients in the morning dosing group received more adjustments in medication dosage. This finding suggests that the greater efficacy observed in the bedtime dosing group is not attributable to higher drug doses.

Patients’ adherence is reported to be affected by the timing of medication administration. Previous studies have shown that patients in the morning dosing group might have better treatment adherence compared with the evening group.^16,31^ In studies such as TIME and the Hygia Chronotherapy Trial, patients were often taking multiple medications, or dosing some in the morning and others at night, which might further highlight the differences in adherence related to dosing timing in those studies.^12,16^ Our present study aimed to address these limitations by using a single-pill combination to improve patient adherence, as recommended by guidelines,^3,4^ strictly fixing the morning and bedtime dosing times, and evaluating and reinforcing treatment adherence during follow-ups and by random investigation calls. Moreover, the convenience of evening dosing might improve adherence in some patients.^32^ The mean (SD) age of our study population was 55.5 (10.6) years, with 75.8% younger than 65 years, meaning most of the population was still working. Young and middle-aged Chinese patients, often busy with work and rushed in the morning, may find it more practical to take their medications in the evening after work.^33^ This might explain why adherence did not differ significantly between the morning and bedtime dosing groups in our study.

Currently, there is no universally accepted definition of nocturnal hypotension according to ABPM; therefore, based on previous studies,^34,35^ we used 2 BPs—90/50 mm Hg and 100/60 mm Hg—as the definitions. We found no significant difference in the incidence of nocturnal hypotension or adverse events (eg, headache, hypotension, and oliguria) between groups, regardless of the comorbidities, which suggested an equal tolerability of bedtime dosing and morning dosing among these populations. These findings provide new evidence supporting the safety of evening medication dosing.

### Strengths and Limitations

The OMAN trial has several strengths. First, we used daily diaries to precisely determine the start and end of the activity or sleep periods for deriving ABPM values (daytime or nighttime), instead of relying on fixed clock hours. Second, titration of the drug regimen during follow-up was based on both ABPM and OBPM, rather than solely OBPM, which provided supplementary information for optimizing hypertension management. Third, to our knowledge, our study is the first multicenter randomized clinical trial to use a long-acting single-pill combination of an angiotensin receptor blocker and a calcium-channel blocker as the initial treatment for an antihypertensive chronotherapy study, with strictly fixed morning and bedtime dosing times.

This study also has several limitations. First, the study included only Chinese participants without cardiovascular disease, so the generalizability of our findings to other ethnic populations or patients with cardiovascular disease remains uncertain. Second, as a supplementary tool to OBPM, the lack of widespread availability of ABPM in clinical practice and its limited use in outcome trials to determine medication titration may limit the generalizability of our findings. Third, adherence to the timing of dosing was participant-reported, which could introduce measurement error. Moreover, the high adherence in this short-term efficacy study may not be representative of long-term outcome trials or clinical settings. Fourth, the results of this study reflect the effect of olmesartan-amlodipine on nighttime BP reduction, and may not be generalizable to other single-pill combinations or antihypertensive drugs in the context of chronotherapy. Fifth, the follow-up period was limited to 3 months, and cardiovascular outcomes were not assessed. The long-term cardiovascular benefits of antihypertensive chronotherapy require further validation through large-scale randomized clinical trials.

## Conclusions

In this randomized clinical trial of antihypertensive chronotherapy, bedtime dosing provided better control of nocturnal BP and improved circadian rhythm, without reducing the efficacy on mean daytime or 24-hour BP or increasing the risk of nocturnal hypotension. These findings support the potential advantages of bedtime administration and offer new evidence to guide future research on antihypertensive chronotherapy.

## Notes

Supplement 1.
Trial Protocol and Statistical Analysis Plan

Supplement 2. **eTable 1.** Inclusion/Exclusion Criteria **eTable 2.** Comparison of Follow-Up and Lost to Follow-Up Patients **eTable 3.** Differences in Changes Between the Morning and Bedtime Dosing Groups in Office BP Values and Ambulatory BP Values in the Intention-to-Treat Population **eTable 4.** Differences in Changes Between the Morning and Bedtime Dosing Groups in Office BP Values and Ambulatory BP Values in the Per-Protocol Population **eTable 5.** Sensitivity Analysis: Multiple Imputation Analysis by Regression Model, ITT Population **eTable 6.** Sensitivity Analysis: Multiple Imputation by Chained Equations, ITT Population **eTable 7.** Differences in Changes Between the Morning and Bedtime Dosing Groups in Office BP Values and Ambulatory BP Values in the Intention-to-Treat Population Using a Linear Mixed-Effects Model **eTable 8.** Differences in Changes Between the Morning and Bedtime Dosing Groups in BP Load **eTable 9.** The Treatment Response Rate for OBPM at Week 4, 8, and 12 **eTable 10.** The Proportions of Patients Receiving Dosage Titration During Follow-Ups and Mean BP Values **eTable 11.** Adverse Events Reported by Participants and the Incidence of Nocturnal Hypotension During the Study **eTable 12.** Adverse Events and the Incidence of Nocturnal Hypotension Stratified by Age **eTable 13.** Adverse Events and the Incidence of Nocturnal Hypotension Stratified by Comorbidities **eTable 14.** The Number of Missing Data Imputed for the ITT Analysis **eFigure 1.** The Mean SBP of Office and ABPM Values at Baseline and During Follow-Ups **eFigure 2.** The Mean DBP of Office and ABPM Values at Baseline and During Follow-Ups **eFigure 3.** Office and ABPM BP Control Rates at Baseline and During Follow-Ups **eFigure 4.** The Distribution of Dipping Status for Morning Dosing and Bedtime Dosing Groups at Week 12 **eFigure 5.** Mean Hourly Ambulatory BP Profiles Illustrating the Overall BP Fluctuations Across All Patients at Baseline and Week 12 for SBP (A) and DBP (B) **eFigure 6.** Differences in Nighttime SBP Between the Morning and Bedtime Dosing Groups at 12 Weeks in Patient Subgroups **eAppendix.** Findings and Responsibilities of the Independent Monitoring Committee

Supplement 3.
Data Sharing Statement
