# Supplementary material for: Morning vs Bedtime Dosing and Nocturnal Blood Pressure Reduction in Patients With Hypertension: The OMAN Randomized Clinical Trial
Source: JAMA Netw Open. 2025 Jul 9;8(7):e2519354. doi: 10.1001/jamanetworkopen.2025.19354 (PMC12242701; doi:10.1001/jamanetworkopen.2025.19354)
Supplement: Supplement 1. — Trial Protocol and Statistical Analysis Plan [file jamanetwopen-e2519354-s001.pdf]

Original trial protocol and statistical analysis plan

Study title: A randomized controlled study for the effects of Olmesartan/Amlodipine administrated in Morning or At Nighttime on nocturnal blood pressure reduction in Chinese patients with mild-moderate essential hypertension.

Original Protocol: Version 2.0 10 January 2022

Introduction

Hypertension is the most common chronic non-communicable disease and the most important risk factor for cardiovascular disease worldwide. Moreover, hypertension significantly increases the risks of atherosclerotic heart disease, stroke, chronic kidney disease, and heart failure. According to the recent China Hypertension Survey, the age-adjusted prevalence of hypertension in Chinese adults aged >18 years is 23.2%, with the estimated number of affected patients exceeding 250 million; however, the blood pressure (BP) control rate is only 16.8%, which is significantly lower than that in the developed countries of Europe and the USA[1]. In 2017 alone, 2.54 million Chinese individuals died as a result of high systolic BP (SBP), with cardiovascular disease being the immediate cause of death in 95.7% of cases[2]. Therefore, effective control of BP is critical in the prevention and treatment of cardiovascular disease and for the reduction of its high mortality rate.

The Patient-Centered Cardiac Event Evaluation Million Population Project, which included 1.7 million community-dwelling residents aged 35–75 years in 31 provinces and autonomous regions in mainland China, found that 81.1% of treated

hypertensive patients took a single anti-hypertensive agent but that the proportion of patients with uncontrolled BP on combination therapy was only 18.6%[3]. This finding suggests that low utilization of combination therapy may be an important reason for the low BP control rate in hypertensive patients in China. The single-pill combination (SPC) is an oral antihypertensive regimen that includes two or more agents. Several clinical trials have demonstrated that SPC is safe and effective in protecting target organs, reducing/offsetting adverse reactions, increasing BP control synergistically, and lowering the incidence of cardiovascular disease and deaths from the disease[4]. Therefore, SPC is recommended as the initial choice in the recently published international hypertension guideline[5]. Olmesartan/amlodipine (OA) is a novel SPC formed by the combination of an angiotensin receptor antagonist and a calcium ion blocker and may effectively lower peripheral vascular resistance and have an antihypertensive effect without affecting glucose or lipid metabolism. Previous large-scale clinical studies have confirmed that OA can significantly reduce BP in hypertensive patients who do not meet their BP target on monotherapy[6, 7]. However, those studies used only office BP as the indicator of efficacy. Therefore, there is a lack of information on the effectiveness of OA in reducing 24-h BP, daytime BP, nighttime BP, and morning BP as well as in restoring the circadian rhythm of BP.

According to recent studies, uncontrolled nocturnal BP is particularly common in hypertensive patients on antihypertensive medication[8], with an incidence rate of 30–60% depending on the study cohort[9–12]. Mean nighttime BP is a better predictor of cardiovascular events than daytime BP or 24-h mean BP[13]. A prospective cohort

study demonstrated that every 20mmHg increase in nighttime BP was associated with a 23% increase in the risk of all-cause mortality and a 36% increase in cardiovascular and cerebrovascular events[14]. Controlling nighttime BP and restoring the circadian rhythm of BP are effective ways of reducing the risk of cardiovascular and cerebrovascular events in hypertensive patients.

However, managing nocturnal BP is challenging in practice. Antihypertensive chronotherapy may provide a new direction for effective management of nocturnal BP. A recently published multicenter, prospective, randomized controlled study from Spain (known as Hygia Chronotherapy Trial) found that taking antihypertensive medication before bedtime significantly reduced the risk of cardiovascular death, myocardial infarction, and major cardiovascular composite events (including coronary revascularization, heart failure, or stroke) in hypertensive patients[15]. However, a two-center randomized, crossover, prospective clinical study performed in London and Thessaloniki suggested that the timing of antihypertensive drug delivery (morning vs. nighttime) did not affect 24-h BP, daytime BP, or nocturnal BP levels in hypertensive patients[16]. Furthermore, the clinical evidence for antihypertensive chronotherapy comes mostly from populations on a single antihypertensive agent in Europe or the USA or from populations with cardiovascular disease. High heterogeneity, including in intervention methods, evaluation indicators, and study populations, between these studies has been noted[17]. Moreover, a systematic review of 21 studies that compared antihypertensive medication taken in the morning with that taken at bedtime indicated that both dosing strategies showed no statistical

difference in 24-h mean BP reduction values[18]. Thus, the clinical benefit of taking antihypertensive drugs before bedtime remains controversial.

Therefore, we planed to initiate this prospective, randomized, open-label, parallel-group clinical study to compare the effects of morning and bedtime administration on nocturnal BP reduction and recovery of circadian rhythm in patients with essential hypertension. The results of this trial are expected to provide useful guidance for optimizing BP control, particularly in Chinese patients with hypertension.

Primary Objective:

The decrease in average nocturnal SBP between baseline and after 12 weeks of the intervention.

Secondary Objective:

24-h ambulatory BP measurements

1. Reductions in 24-h, daytime and morning average SBP and diastolic BP (DBP) at weeks 4 and 12.

2. Average 24-h, daytime, nocturnal SBP and DBP control rates at weeks 4 and 12.

3. Changes of proportion of non-dipper, and changes in BP load.

Office BP measurements

(1) The reduction in average SBP and DBP in the clinic at weeks 4, 8, and 12.

(2) Office BP control rates.

(3) The treatment response rate (SBP <140 mmHg or a decrease≥20 mmHg and DBP <90 mmHg or a decrease≥10 mmHg) at weeks 4, 8, and 12.

Proportion of patients requiring intensive treatment.

Study Design:

This prospective, multicenter, randomized, open-label, parallel-group clinical trial will be performed at 20 hospitals across Sichuan in China. However, in practice, 15 sites fulfilled the study protocol and conducted the study.

Study participants will be randomized in a 1:1 ratio to a morning (6–10 am) or a bedtime (6–10 pm) administration group. Initially, each participant will receive one tablet of OA (20/5 mg) daily for 4 weeks and will be then followed up at 4-week intervals for a total of 12 weeks. The OA dosage will be adjusted according to the results of ABPM and OBPM during follow-up. Controlled BP is defined as a 24-h mean BP <130/80 mmHg, daytime mean BP <135/85 mmHg, nighttime mean BP <120/70 mmHg, and OBPM <140/90 mmHg. The OA dosage will be increased to 1.5 tablets/day in patients who are found to have masked or sustained uncontrolled hypertension at the initial follow-up visit. If BP remains uncontrolled after a further 4 weeks, the OA dosage can be increased further to 2 tablets/day. All participants are instructed to discontinue their preexisting antihypertensive medications for at least 2 weeks before starting the trial intervention. Adherence with medication will be recorded at each follow-up visit. Concomitant use of other antihypertensive drugs other than OA will be prohibited during this study.

Study Duration:

The study started in June 2022 and is scheduled to conclude in September 2023. However, due to the longer preparation of two sites, the follow-ups of patients were

lasted until April 2024.

Inclusion Criteria:

1. Patients with essential hypertension, aged 18–75 years, of either sex, with regularly scheduled activities and work and rest durations.

2. Patients who have not previously received antihypertensive treatment or stopped using antihypertensive agents for 2 weeks before starting the trial medication.

3. Able to provide a signed informed consent form and willingness to attend for follow-up in a timely manner.

Exclusion Criteria:

1. Extreme dipper (nocturnal BP >20% lower than daytime BP).

2. Pregnancy, planning a pregnancy, or breastfeeding.

3. Renal artery stenosis.

4. Hyperkalemia (serum potassium >5.5 mmol/L), chronic renal insufficiency (creatinine >265μmol/L).

5. History of or progressing toward malignant hypertension, hypertensive emergency, hypertensive crisis, or hypertensive encephalopathy during the 2-week antihypertensive drug withdrawal phase and enrollment.

6. History of drug use or other causes of angioedema or a history of hypersensitivity to angiotensin receptor antagonists, angiotensin-converting enzyme inhibitors, or renin inhibitors.

7. History of alcohol or drug abuse.

8. Working at night or shift work.

9. Cardiovascular disease, such as unstable angina, heart failure, life-threatening arrhythmias, atrial fibrillation, renal failure, hypertrophic cardiomyopathy, and grade III–IV retinopathy.

10. Unable to tolerate ambulatory BP measurement or participate in clinical research.

11. History of allergy to amlodipine.

12. History of hemorrhagic stroke.

13. Participating in or planning to participate in other trials.

14. Poor compliance.

Collection of medical data and quality control:

This research is initiated by Professor Xiaoping Chen at the Hypertension Center, West China Hospital, Sichuan University. Trained physicians, nurses, and clinical research coordinators (CRCs) are required to keep medical records and collect data in accordance with the study protocol.

Baseline data on demographic characteristics, height, and body weight will be collected, and blood biochemistry tests will be performed. Office BP will be measured using calibrated electronic sphygmomanometers (HBP-9020, Omron Corp., Kyoto, Japan) according to the guidelines for the management of hypertension[19, 20] (However, due to budget constraints, we decided to switch to the Omron HBP-1100-E (Omron Healthcare Co., Ltd, Kyoto, Japan) before the trial commenced). To obtain accurate BP data, all participants are requested to rest for 5 min before the measurement. SBP and DBP are obtained three times on the right arm in a sitting position, with the average of the last two values calculated as mean office BP. ABPM

readings are obtained from the non-dominant arm at baseline and weeks 4 and 12 using a TM2430 BP monitor (A&D Inc., Tokyo, Japan). During monitoring, BP is measured at 20 min intervals throughout the day (usually from 6 am to 10 pm) and then at 30 min intervals at night (usually from 10 pm to 6 am). Valid recordings will span >20 h, including at least 20 during the day and 10 during the night[21, 22]. A diary card is issued to each participant so that they can record their schedule in detail, including the times they go to bed and wake up. The mean 24-h BP was calculated as the average of all successful values. Nighttime BP was defined as the mean BP recorded between falling asleep and waking up. Daytime BP was defined as the mean value of all other readings. Morning BP was defined as the average BP within 2 hours after waking up[23]. Circadian rhythms were categorized according to the extent of nocturnal fall in BP: extreme dipper (>20% fall); dipper (10-20% fall); non-dipper (0 to <10% fall); and reverse dipper (<0% fall). The nocturnal fall in BP was calculated as (Daytime BP-Nighttime BP)/Daytime BP*100%. BP load was defined as the percentage of readings above the normal value during the daytime, nighttime, or 24-h period.

The medical officer assigned to each study participant is alerted to remind the participant to attend all interventions and assessments in an effort to minimize the dropout rate. Participants and their caregivers also have direct access to a hotline during office hours if they have any concerns or problems. All original research data are entered into the electronic data capture system, namely, the Red Shine Chronic Disease Management System developed by the Hypertension Center at West China

Hospital (http://47.108.235.3:3003/#/login). All study-related information is stored securely in the system and all information concerning individual study participants is stored in locked filing cabinets in areas with limited access. A training session on the study protocol was provided before initiation of the study at each participating site to ensure that the quality of this clinical trial is satisfactory. On-site monitoring at the beginning, middle, and the end of the study is performed by assigned clinical researchers to ensure complete compliance with the study protocol. A project specialist has been assigned to this trial for data monitoring, examination, and cleaning. This specialist will perform the interim analyses, the frequency of which will be decided by the specialist in consultation with the principal investigator. The specialist will advise the principal investigator of the interim findings if necessary. After data cleaning is completed, the data managers and statisticians will conduct a final review of unresolved data issues, discuss the division of datasets together according to the statistical analysis plan, and examine any reports of serious adverse events. A statistical analysis is to be conducted halfway through and at the end of the trial. The subjects will be discontinued from follow-up if individual participants report any serious adverse effects during the intervention or a significant deterioration in their mental or physical state.

Statistical Method:

The study population is divided into a morning administration group and a bedtime administration group. Quantitative variables will be summarized as the mean ± SD or as the median [interquartile range (IQR)] depending on the type of the original data.

Qualitative variables will be presented as the absolute and relative frequency. Quantitative variables will be compared between the two study groups using the t-test or Mann-Whitney U test and qualitative variables using the chi-squared test or Fisher's exact test.

All analyses will be based on the intention-to-treat (ITT) and the per-protocol (PP) populations. The primary objective of the study will be analyzed using an analysis of covariance (ANCOVA) model with appropriate baseline adjustments. The ANCOVA model will also be used to evaluate the other secondary objectives. Mean differences or odds ratios will be determined across groups along with their 95% confidence intervals.All statistical analyses will be performed using SPSS version 26.0 (IBM Corp., Armonk, NY, USA). The significance level will be set to 5% for all statistical tests.

The process for handling missing data is generally divided into the deletion of cases with missing data and imputation of missing data. The commonly used methods include mean imputation, last observation carried forward, and multiple imputation. We will deal with the missing data depending on the circumstances under the guidance of the Department of Clinical Research Management at West China Hospital.

Sample Size Consideration:

Considering the lack of published studies on the nocturnal systolic BP reduction after treatment with a fixed-dose OA combination, we referred to the study on BP control reported by Wang et al. [24], in which nocturnal systolic BP was reduced by−14.7 ±

11.6 mmHg in patients who received a fixed-dose combination of valsartan/amlodipine. We also collected and analyzed the medical data for 50 patients who had received OA at our center. Our preliminary analysis revealed that the standard deviation (SD) for nocturnal SBP reduction was 12 mmHg in subjects who took OA for 3 months. According to the above analysis, we set SD as 12 when calculating the sample size. In terms of the endpoint, a superiority margin of 3mmHg was considered a clinically meaningful BP reduction on the basis of the observed decreases in cardiovascular morbidity with small reductions in systolic BP (2-5mmHg) by pharmacological therapy. After discussion with clinical experts, we selected 3 mmHg as the superiority margin. Hence, while the significance level was set to 0.05 (two-sided) and the power to 85%, 289/289 cases would be needed for each category. Assuming a 20% dropout rate, each study group would comprise at least 360 patients, for a total of 720 cases. PASS version 15.0 was used to calculate the sample size.

Reference:

1. Wang Z, Chen Z, Zhang L, Wang X, Hao G, Zhang Z, Shao L, Tian Y, Dong Y, Zheng C, et al. Status of hypertension in China: results from the China hypertension survey, 2012–2015. Circulation. 2018;137(22):2344–56.

2. Zhou M, Wang H, Zeng X, Yin P, Zhu J, Chen W, Li X, Wang L, Wang L, Liu Y, et al. Mortality, morbidity, and risk factors in China and its provinces, 1990–2017: a systematic analysis for the Global Burden of Disease Study 2017. Lancet. 2019;394(10204):1145–58.

3. Lu J, Lu Y, Wang X, Li X, Linderman GC, Wu C, Cheng X, Mu L, Zhang H, Liu J,

et al. Prevalence, awareness, treatment, and control of hypertension in China: data from 1·7 million adults in a population-based screening study (China PEACE Million Persons Project). Lancet. 2017;390(10112):2549–58.

4. Parati G, Kjeldsen S, Coca A, Cushman WC, Wang J. Adherence to single-pill versus free-equivalent combination therapy in hypertension: a systematic review and meta-analysis. Hypertension. 2021;77(2):692–705.

5. Unger T, Borghi C, Charchar F, Khan NA, Poulter NR, Prabhakaran D, Ramirez A, Schlaich M, Stergiou GS, Tomaszewski M, et al. 2020 International Society of Hypertension Global Hypertension Practice Guidelines. Hypertension. 2020;75(6):1334–57.

6. Chrysant SG, Melino M, Karki S, Lee J, Heyrman R. The combination of olmesartan medoxomil and amlodipine besylate in controlling high blood pressure: COACH, a randomized, double-blind, placebo-controlled, 8-week factorial efficacy and safety study. Clin Ther. 2008;30(4):587–604.

7. Zhu J-R, Zhang S-Y, Gao P-J. Efficacy and safety of olmesartan medoxomil/amlodipine fixed-dose combination for hypertensive patients uncontrolled with monotherapy. Arch Pharm Res. 2014;37(12):1588–98.

8. Tadic M, Cuspidi C, Grassi G, Mancia G. Isolated nocturnal hypertension: what do we know and what can we do? Integr Blood Press Control. 2020;13:63–9.

9. Cuspidi C, Facchetti R, Bombelli M, Sala C, Tadic M, Grassi G, Mancia G. Is night-time hypertension worse than daytime hypertension? A study on cardiac damage in a general population: the PAMELA study. J Hypertens. 2017;35(3):506–12.

10. Androulakis E, Papageorgiou N, Chatzistamatiou E, Kallikazaros I, Stefanadis C, Tousoulis D. Improving the detection of preclinical organ damage in newly diagnosed hypertension: nocturnal hypertension versus non-dipping pattern. J Hum Hypertens. 2015;29(11):689–95.

11. Ogedegbe G, Spruill TM, Sarpong DF, Agyemang C, Chaplin W, Pastva A, Martins D, Ravenell J, Pickering TG. Correlates of isolated nocturnal hypertension and target organ damage in a population-based cohort of African Americans: the Jackson Heart Study. Am J Hypertens. 2013;26(8):1011–6.

12. Wang C, Deng W-J, Gong W-Y, Zhang J, Zhang Q-Z, Ye ZC, Lou T. Nocturnal hypertension correlates better with target organ damage in patients with chronic kidney disease than a nondipping pattern. J Clin Hypertens (Greenwich). 2015;17(10):792–801.

13. Boggia J, Li Y, Thijs L, Hansen TW, Kikuya M, Björklund-Bodegård K, Richart T, Ohkubo T, Kuznetsova T, Torp-Pedersen C, et al. Prognostic accuracy of day versus night ambulatory blood pressure: a cohort study. Lancet. 2007;370(9594):1219–29.

14. Yang W-Y, Melgarejo JD, Thijs L, Zhang Z-Y, Boggia J, Wei F-F, Hansen TW, Asayama K, Ohkubo T, Jeppesen J, et al. Association of office and ambulatory blood pressure with mortality and cardiovascular outcomes. JAMA. 2019;322(5):409–20.

15. Hermida RC, Crespo JJ, Domínguez-Sardiña M, Otero A, Moyá A, Ríos MT, Sineiro E, Castiñeira MC, Callejas PA, Pousa L, et al. Bedtime hypertension treatment improves cardiovascular risk reduction: the Hygia Chronotherapy Trial. Eur Heart J. 2020;41(48):4565–76.

16. Poulter NR, Savopoulos C, Anjum A, Apostolopoulou M, Chapman N, Cross M, Falaschetti E, Fotiadis S, James RM, Kanellos I, et al. Randomized crossover trial of the impact of morning or evening dosing of antihypertensive agents on 24-hour ambulatory blood pressure. Hypertension.
2018;72(4):870–3.

17. Thoonkuzhy C, Rahman M. New insights on chronotherapy in hypertension: is timing everything? Curr Hypertens Rep. 2020;22(4):32.

18. Zhao P, Xu P, Wan C, Wang Z. Evening versus morning dosing regimen drug therapy for hypertension. Cochrane Database Syst Rev. 2011;2011(10):CD004184.

19. Mancia G, Kreutz R, Brunström M, et al. 2023 ESH Guidelines for the management of arterial hypertension The Task Force for the management of arterial hypertension of the European Society of Hypertension: Endorsed by the International Society of Hypertension (ISH) and the European Renal Association (ERA). J Hypertens 2023; 41(12): 1874-2071.

20. Revision Committee of guidelines on the management of hypertension in China. 2018 Chinese guidelines for the management of hypertension. Chin J Cardiovasc Med 2019; 24(1): 24–56.

21. Kario K, Hoshide S, Chia Y-C, et al. Guidance on ambulatory blood pressure monitoring: A statement from the HOPE Asia Network. J Clin Hypertens (Greenwich) 2021; 23(3): 411-421.

22. Writing Group of the 2020 Chinese Hypertension League Guidelines on Ambulatory Blood Pressure Monitoring. 2020 Chinese Hypertension League

Guidelines on Ambulatory Blood Pressure Monitoring. Chinese Circulation Journal. 2021; 36(04): 313-328.

23. Kario K, Wang JG, Chia YC, et al. The HOPE Asia network 2022 up-date consensus statement on morning hypertension management. J Clin Hypertens (Greenwich). 2022;24(9):1112-1120.

24. Wang K-L, Yu W-C, Lu T-M, Chen L-C, Leu H-B, Chiang C-E. Amlodipine/valsartan fixed-dose combination treatment in the management of hypertension: a double-blind, randomized trial. J Chin Med Assoc. 2020;83(10):900–5.
