# Supplementary material for: Morning vs Bedtime Dosing and Nocturnal Blood Pressure Reduction in Patients With Hypertension: The OMAN Randomized Clinical Trial
Source: JAMA Netw Open. 2025 Jul 9;8(7):e2519354. doi: 10.1001/jamanetworkopen.2025.19354 (PMC12242701; doi:10.1001/jamanetworkopen.2025.19354)
Supplement: Supplement 2. — eTable 1. Inclusion/Exclusion Criteria eTable 2. Comparison of Follow-Up and Lost to Follow-Up Patients eTable 3. Differences in Changes Between the Morning and Bedtime Dosing Groups in Office BP Values and Ambulatory BP Values in the Intention-to-Treat Population eTable 4. Differences in Changes Between the Morning and Bedtime Dosing Groups in Office BP Values and Ambulatory BP Values in the Per-Protocol Population eTable 5. Sensitivity Analysis: Multiple Imputation Analysis by Regression Model, ITT Population eTable 6. Sensitivity Analysis: Multiple Imputation by Chained Equations, ITT Population eTable 7. Differences in Changes Between the Morning and Bedtime Dosing Groups in Office BP Values and Ambulatory BP Values in the Intention-to-Treat Population Using a Linear Mixed-Effects Model eTable 8. Differences in Changes Between the Morning and Bedtime Dosing Groups in BP Load eTable 9. The Treatment Response Rate for OBPM at Week 4, 8, and 12 eTable 10. The Proportions of Patients Receiving Dosage Titration During Follow-Ups and Mean BP Values eTable 11. Adverse Events Reported by Participants and the Incidence of Nocturnal Hypotension During the Study eTable 12. Adverse Events and the Incidence of Nocturnal Hypotension Stratified by Age eTable 13. Adverse Events and the Incidence of Nocturnal Hypotension Stratified by Comorbidities eTable 14. The Number of Missing Data Imputed for the ITT Analysis eFigure 1. The Mean SBP of Office and ABPM Values at Baseline and During Follow-Ups eFigure 2. The Mean DBP of Office and ABPM Values at Baseline and During Follow-Ups eFigure 3. Office and ABPM BP Control Rates at Baseline and During Follow-Ups eFigure 4. The Distribution of Dipping Status for Morning Dosing and Bedtime Dosing Groups at Week 12 eFigure 5. Mean Hourly Ambulatory BP Profiles Illustrating the Overall BP Fluctuations Across All Patients at Baseline and Week 12 for SBP (A) and DBP (B) eFigure 6. Differences in Nighttime SBP Between the Morning and Bedtime [file jamanetwopen-e2519354-s002.pdf]

Supplementary Online Content

Ye R, Yang X, Zhang X, et al. Morning vs bedtime dosing and nocturnal blood pressure reduction in hypertension: the OMAN randomized clinical trial. *JAMA Netw Open.* 2025;8(7):e2519354. doi:10.1001/jamanetworkopen.2025.19354

eTable 1. Inclusion/Exclusion Criteria
eTable 2. Comparison of Follow-Up and Lost to Follow-Up Patients
eTable 3. Differences in Changes Between the Morning and Bedtime Dosing Groups in Office BP Values and Ambulatory BP Values in the Intention-to-Treat Population
eTable 4. Differences in Changes Between the Morning and Bedtime Dosing Groups in Office BP Values and Ambulatory BP Values in the Per-Protocol Population
eTable 5. Sensitivity Analysis: Multiple Imputation Analysis by Regression Model, ITT Population
eTable 6. Sensitivity Analysis: Multiple Imputation by Chained Equations, ITT Population
eTable 7. Differences in Changes Between the Morning and Bedtime Dosing Groups in Office BP Values and Ambulatory BP Values in the Intention-to-Treat Population Using a Linear Mixed-Effects Model
eTable 8. Differences in Changes Between the Morning and Bedtime Dosing Groups in BP Load
eTable 9. The Treatment Response Rate for OBPM at Week 4, 8, and 12
eTable 10. The Proportions of Patients Receiving Dosage Titration During Follow-Ups and Mean BP Values
eTable 11. Adverse Events Reported by Participants and the Incidence of Nocturnal Hypotension During the Study
eTable 12. Adverse Events and the Incidence of Nocturnal Hypotension Stratified by Age
eTable 13. Adverse Events and the Incidence of Nocturnal Hypotension Stratified by Comorbidities
eTable 14. The Number of Missing Data Imputed for the ITT Analysis
eFigure 1. The Mean SBP of Office and ABPM Values at Baseline and During Follow-Ups
eFigure 2. The Mean DBP of Office and ABPM Values at Baseline and During Follow-Ups
eFigure 3. Office and ABPM BP Control Rates at Baseline and During Follow-Ups
eFigure 4. The Distribution of Dipping Status for Morning Dosing and Bedtime Dosing Groups at Week 12
eFigure 5. Mean Hourly Ambulatory BP Profiles Illustrating the Overall BP Fluctuations Across All Patients at Baseline and Week 12 for SBP (A) and DBP (B)
Figure 6. Differences in Nighttime SBP Between the Morning and Bedtime Dosing Groups at 12 Weeks in Patient Subgroups
eAppendix. Findings and Responsibilities of the Independent Monitoring Committee

This supplementary material has been provided by the authors to give readers additional information about their work.

© 2025 Ye R et al. *JAMA Network Open.*

eTable 1. Inclusion/Exclusion Criteria

**Inclusion Criteria**
1. Patients with essential hypertension (elevated ABPM readings of 24-h BP ⩾ 130/80mmHg, or daytime BP ⩾135/85 mmHg, or nighttime ⩾120/70 mmHg, with elevated office BP (⩾140/90mmHg) during screening period), aged 18-75 years, of either sex, with regularly scheduled activities and work and rest durations.
2. Patients who have not previously received antihypertensive treatment or stopped using antihypertensive agents, or drugs may affect BP (such as SGLT2i) for at least 2 weeks before the trial.
3. Patients who are able to provide a signed informed consent form and willing to attend for follow-up in a timely manner.
**Exclusion criteria**
1. Normal ABPM readings of 24-h BP<130/80mmHg, daytime BP <135/85 mmHg, and nighttime <120/70 mmHg, but elevated office BP (≥140/90mmHg).
2. Extreme dipper [(daytime BP-nighttime BP)/daytime BP*100% >20%].
3. Pregnancy, planning a pregnancy, or breastfeeding.
4. Renal artery stenosis.
5. Hyperkalemia (serum potassium >5.5 mmol/L), and chronic renal insufficiency (creatinine >265μmol/L).
6. History of or progression toward hypertensive urgencies or emergencies (office BP >180/120mmHg) during the 2-week antihypertensive drug withdrawal phase and enrollment.
7. History of drug use or other causes of angioedema or a history of hypersensitivity to angiotensin receptor antagonists, angiotensin-converting enzyme inhibitors, or renin inhibitors.
8. History of alcohol or drug abuse.
9. Working at night or shift work.
10. Cardiovascular disease, such as unstable angina, heart failure, life-threatening arrhythmias, atrial fibrillation, renal failure, hypertrophic cardiomyopathy, and grade III–IV retinopathy.
11. Unable to tolerate ambulatory BP measurement or participate in clinical research.
11. History of allergy to amlodipine.
12. History of hemorrhagic stroke.
13. Participation in or planning to participate in other trials.
14. Poor compliance.

© 2025 Ye R et al. *JAMA Network Open.*

eTable 2. Comparison of Follow-Up and Lost to Follow-Up Patients

**Variables**	**Follow-up (n=607)**	**Lost of follow-up (n=113)**	**P value**
Age, mean (SD), y	55.40±10.57	56.19±10.88	.47
Sex			.56
Male	342 (56.3)	67 (59.3)	
Female	265 (43.7)	46 (40.7)	
Total cholesterol, mean (SD), mg/dL	196.73 (43.70)	192.97 (36.32)	.33
LDL-cholesterol, mean (SD), mg/dL	114.22 (35.55)	117.08 (32.76)	.40
HDL-cholesterol, mean (SD), mg/dL	51.03 (16.56)	50.13 (11.73)	.49
TG, median (IQR), mg/dL	142.56 (104.48, 207.20)	146.10 (97.84, 192.25)	.81
Plasma glucose, median (IQR), mg/dL	96.59 (88.30, 109.74)	98.93 (91.63, 108.31)	.22
Baseline BP, mean (SD), mmHg			
Office SBP	154.13 (12.31)	155.37 (12.58)	.33
Office DBP	94.83 (10.69)	94.94 (10.70)	.92
Office PP	78.28 (10.79)	78.37 (12.45)	.94
24-h SBP	147.63 (10.99)	148.52 (11.18)	.43
24-h DBP	91.58 (8.91)	90.89 (9.92)	.46
24-h PP	75.99 (9.61)	76.34 (10.45)	.73

SI conversion factors: To convert cholesterol to mmol/L, multiply by 0.0259; To convert triglyceride to mmol/L, multiply by 0.0113; To convert glucose to mmol/L, multiply by 0.0555.

Abbreviations: BP, blood pressure; SBP, systolic blood pressure; DBP, diastolic blood pressure; PP, pulse pressure; HDL-C, high-density lipoprotein cholesterol; LDL-C, low-density lipoprotein cholesterol; TG, triglyceride.

© 2025 Ye R et al. *JAMA Network Open.*

eTable 3. Differences in Changes Between the Morning and Bedtime Dosing Groups in Office BP Values and Ambulatory BP Values in the Intention-to-Treat Population

	Morning dosing (n=358)	Bedtime dosing (n=362)	Difference	Adjusted difference	P-value
**SBP, mean (SD), mmHg**					
**Office**					
Changes at 4 weeks	-20.09 (17.96)	-21.88 (18.31)	-1.79 (-4.45, 0.86)	-1.86 (-4.21, 0.50)	.12
Changes at 8 weeks	-23.46 (16.88)	-24.91 (18.29)	-1.46 (-4.03, 1.12)	-1.53 (-3.73, 0.68)	.18
Changes at 12 weeks	-25.97 (19.82)	-27.81 (17.88)	-1.84 (-4.61, 0.92)	-1.92 (-4.22, 0.38)	.10
**Daytime**					
Changes at 4 weeks	-17.20 (14.45)	-16.80 (14.52)	0.40 (-1.72, 2.52)	0.13 (-1.91, 2.17)	.90
Changes at 12 weeks	-22.07 (14.75)	-22.73 (14.43)	-0.66 (-2.79, 1.48)	-1.05 (-3.01, 0.91)	.29
**Nighttime**					
Changes at 4 weeks	-17.51 (15.93)	-19.54 (15.62)	-2.03 (-4.34, 0.28)	-2.05 (-4.18, 0.09)	.06
Changes at 12 weeks	-22.30 (16.82)	-25.32 (14.96)	-3.02 (-5.35, -0.69)	-3.04 (-5.11, -0.97)	.004
**24-h**					
Changes at 4 weeks	-17.39 (13.71)	-17.95 (13.82)	-0.56 (-2.58, 1.45)	-0.70 (-2.64, 1.24)	.48
Changes at 12 weeks	-22.16 (14.01)	-23.63 (13.51)	-1.47 (-3.48, 0.55)	-1.66(-3.51, 0.19)	.08
**Morning**					
Changes at 4 weeks	-17.09 (19.23)	-18.37 (19.14)	-1.28 (-4.08, 1.53)	-1.51 (-4.09, 1.08)	.25
Changes at 12 weeks	-21.52 (19.18)	-24.32 (18.73)	-2.80 (-5.57, -0.03)	-3.05 (-5.56, -0.54)	.02
**DBP, mean (SD), mmHg**					
**Office**					
Changes at 4 weeks	-11.45 (12..30)	-12.68 (12.61)	-1.23 (-3.05, 0.59)	-0.96 (-2.47, 0.56)	.22
Changes at 8 weeks	-13.88 (12.35)	-15.55 (12.70)	-1.67 (-3.51, 0.16)	-1.38 (-2.86, 0.10)	.07
Changes at 12 weeks	-14.77 (14.00)	-16.47 (12.94)	-1.71 (-3.68, 0.26)	-1.38 (-2.92, 0.17)	.08

© 2025 Ye R et al. *JAMA Network Open.*

	Morning dosing (n=358)	Bedtime dosing (n=362)	Difference	Adjusted difference	P-value
**Daytime**					
Changes at 4 weeks	-10.69 (9.45)	-10.67 (9.06)	0.02 (-1.33, 1.37)	0.01 (-1.18, 1.20)	.98
Changes at 12 weeks	-13.46 (9.89)	-13.95 (9.15)	-0.49 (-1.88, 0.91)	-0.50 (-1.70, 0.71)	.42
**Nighttime**					
Changes at 4 weeks	-10.41 (9.98)	-11.54 (9.82)	-1.12 (-2.57, 0.33)	-0.96 (-2.22, 0.31)	.14
Changes at 12 weeks	-13.21 (10.92)	-14.83 (10.13)	-1.61 (-3.15, -0.06)	-1.42 (-2.76, -0.09)	.04
**24-h**					
Changes at 4 weeks	-10.67 (8.71)	-11.09 (8.59)	-0.42 (-1.68, 0.85)	-0.33 (-1.45, 0.78)	.56
Changes at 12 weeks	-13.40 (9.42)	-14.30 (8.79)	-0.89 (-2.23, 0.44)	-0.80 (-1.96, 0.36)	.18
**Morning**					
Changes at 4 weeks	-10.14 (13.42)	-12.01 (12.63)	-1.87 (-3.77, 0.04)	-1.31 (-2.93, 0.31)	.11
Changes at 12 weeks	-12.21 (13.92)	-15.52 (13.56)	-3.31 (-5.32, -1.30)	-2.72 (-4.44, -1.01)	.002

© 2025 Ye R et al. *JAMA Network Open.*

eTable 4. Differences in Changes Between the Morning and Bedtime Dosing Groups in Office BP Values and Ambulatory BP Values in the Per-Protocol Population

	Morning dosing (n=298)	Bedtime dosing (n=309)	Difference	Adjusted difference	P-value
**SBP, mean (SD), mmHg**					
**Office**					
Changes at 4 weeks	-21.76 (17.66)	-23.79 (17.37)	-2.03 (-4.82, 0.76)	-1.67 (-4.10, 0.76)	.18
Changes at 8 weeks	-25.38 (16.19)	-27.14 (17.05)	-1.76 (-4.41, 0.90)	-1.36 (-3.53, 0.81)	.22
Changes at 12 weeks	-28.05 (19.41)	-30.29 (16.35)	-2.24 (-5.10, 0.62)	-1.79 (-4.05, 0.47)	.12
**Daytime**					
Changes at 4 weeks	-19.06 (14.20)	-18.13 (14.67)	0.93 (-1.38, 3.23)	0.52 (-1.66, 2.70)	.64
Changes at 12 weeks	-24.62 (13.81)	-25.05 (13.64)	-0.43 (-2.62, 1.76)	-1.03 (-2.93, 0.87)	.29
**Nighttime**					
Changes at 4 weeks	-19.15 (15.58)	-21.14 (15.21)	-1.99 (-4.44, 0.47)	-1.90 (-4.09, 0.29)	.09
Changes at 12 weeks	-24.50 (15.89)	-28.00 (13.43)	-3.51 (-5.85, -1.16)	-3.40 (-5.32, -1.49)	.001
**24-h**					
Changes at 4 weeks	-19.22 (13.31)	-19.40 (13.75)	-0.17 (-2.33, 1.99)	-0.38 (-2.41, 1.65)	.72
Changes at 12 weeks	-24.63 (12.86)	-26.05 (12.45)	-1.41 (-3.43, 0.61)	-1.70 (-3.43, 0.03)	.05
**Morning**					
Changes at 4 weeks	-18.77 (19.12)	-19.81 (19.17)	-1.04 (-4.10, 2.01)	-1.34 (-4.11, 1.44)	.35
Changes at 12 weeks	-23.92 (18.69)	-26.76 (18.24)	-2.84 (-5.79, 0.10)	-3.16 (-5.76, -0.57)	.02
**DBP, mean (SD), mmHg**					
**Office**					
Changes at 4 weeks	-12.55 (12.42)	-13.59 (12.33)	-1.04 (-3.02, 0.93)	-0.70 (-2.26, 0.86)	.38
Changes at 8 weeks	-15.18 (12.37)	-16.85 (12.14)	-1.67 (-3.63, 0.28)	-1.31 (-2.77, 0.16)	.08
Changes at 12 weeks	-15.97 (14.28)	-17.86 (12.50)	-1.89 (-4.03, 0.25)	-1.47 (-3.04, 0.09)	.06

© 2025 Ye R et al. *JAMA Network Open.*

	Morning dosing (n=298)	Bedtime dosing (n=309)	Difference	Adjusted difference	P-value
**Daytime**					
Changes at 4 weeks	-11.86 (9.42)	-11.55 (9.03)	0.31 (-1.16, 1.78)	0.22 (-1.02, 1.46)	.63
Changes at 12 weeks	-15.03 (9.53)	-15.41 (8.59)	-0.37 (-1.82, 1.07)	-0.47 (-1.63, 0.68)	.42
**Nighttime**					
Changes at 4 weeks	-11.43 (9.81)	-12.38 (9.61)	-0.95 (-2.49, 0.60)	-0.91 (-2.20, 0.38)	.17
Changes at 12 weeks	-14.61 (10.64)	-16.37 (9.45)	-1.75 (-3.36, -0.15)	-1.71 (-3.01 -0.42)	.009
**24-h**					
Changes at 4 weeks	-11.81 (8.56)	-11.97 (8.46)	-0.15 (-1.51, 1.20)	-0.18 (-1.32, 0.96)	.76
Changes at 12 weeks	-14.93 (9.03)	-15.78 (8.13)	-0.85 (-2.22, 0.52)	-0.87 (-1.97, 0.22)	.12
**Morning**					
Changes at 4 weeks	-10.87 (13.66)	-12.58 (12.75)	-1.70 (-3.81, 0.40)	-1.06 (-2.80, 0.68)	.23
Changes at 12 weeks	-13.40 (14.09)	-16.78 (13.56)	-3.39 (-5.59, -1.18)	-2.71 (-4.52, -0.89)	.004

© 2025 Ye R et al. *JAMA Network Open.*

eTable 5. Sensitivity Analysis: Multiple Imputation Analysis by Regression Model, ITT Population

	Morning dosing	Bedtime dosing	Adjusted difference	P-value
**SBP changes at 12-week, mean (SD), mmHg**				
Office	-28.27 (18.60)	-29.61 (16.34)	-1.42 (-3.41, 0.56)	.16
Daytime	-24.16 (13.34)	-24.58 (13.16)	-0.88 (-2.55, 0.79)	.30
Nighttime	-24.62 (15.71)	-27.33 (13.30)	-2.74(-4.45, -1.03)	.002
24-h	-24.31 (12.55)	-25.56 (11.97)	-1.48 (-3.00, 0.04)	.06
Morning	-23.58 (17.78)	-26.40 (17.56)	-3.09(-5.34, -0.83)	.007
**DBP changes at 12-week, mean (SD), mmHg**				
Office	-15.94 (13.47)	-17.43 (12.31)	-1.14 (-2.50, 0.22)	.10
Daytime	-14.68 (9.17)	-14.96 (8.35)	-0.29 (-1.31, 0.73)	.58
Nighttime	-14.52 (10.38)	-15.95 (9.31)	-1.23 (-2.38, -0.08)	.04
24-h	-14.62 (8.72)	-15.34 (7.89)	-0.62 (-1.59, 0.36)	.21
Morning	-13.29 (13.38)	-16.42 (13.13)	-2.49 (-4.06, -0.92)	.002

© 2025 Ye R et al. *JAMA Network Open.*

eTable 6. Sensitivity Analysis: Multiple Imputation by Chained Equations, ITT Population

	Morning dosing	Bedtime dosing	Adjusted difference	P-value
**SBP changes at 12-week, mean (SD), mmHg**				
Office	-28.54 (18.78)	-30.03 (16.79)	-1.56 (-3.60, 0.48)	.13
Daytime	-24.77 (13.74)	-24.92 (13.77)	-0.63 (-2.35, 1.10)	.48
Nighttime	-25.17 (16.40)	-27.81 (14.23)	-2.68 (-4.48, -0.87)	.004
24-h	-24.69 (13.02)	-25.65 (12.79)	-1.20 (-2.80, 0.40)	.14
Morning	-23.76 (18.83)	-26.65 (18.60)	-3.17 (-5.56, -0.79)	.009
**DBP changes at 12-week, mean (SD), mmHg**				
Office	-15.98 (14.22)	-17.69 (12.83)	-1.35 (-2.80, 0.10)	.07
Daytime	-14.88 (9.28)	-15.16 (8.49)	-0.29 (-1.33, 0.75)	.58
Nighttime	-14.65 (10.96)	-16.19 (9.87)	-1.32 (-2.55, -0.09)	.04
24-h	-14.91 (9.02)	-15.72 (8.50)	-0.70 (-1.71, 0.32)	.18
Morning	-12.98 (13.63)	-16.16 (13.62)	-2.58 (-4.26, -0.90)	.003

© 2025 Ye R et al. *JAMA Network Open.*

eTable 7. Differences in Changes Between the Morning and Bedtime Dosing Groups in Office BP Values and Ambulatory BP Values in the Intention-to-Treat Population Using a Linear Mixed-Effects Model

	Morning dosing (n=358)	Bedtime dosing (n=362)	Adjusted difference	P-value
**SBP changes at 12-week, mean (SD), mmHg**				
Office	-25.97 (19.82)	-27.81 (17.88)	-1.89 (-3.96, 0.17)	.072
Daytime	-22.07 (14.75)	-22.73 (14.43)	-0.88 (-2.55, 0.79)	.30
Nighttime	-22.30 (16.82)	-25.32 (14.96)	-3.03 (-4.81, -1.26)	.001
24-h	-22.16 (14.01)	-23.63 (13.51)	-1.58 (-3.16, 0.00)	.05
Morning	-21.52 (19.18)	-24.32 (18.73)	-2.96 (-4.98, -0.94)	.004
**DBP changes at 12-week, mean (SD), mmHg**				
Office	-14.77 (14.00)	-16.47 (12.94)	-1.37 (-2.74, 0.00)	.05
Daytime	-13.46 (9.89)	-13.95 (9.15)	-0.49 (-1.52, 0.53)	.35
Nighttime	-13.21 (10.92)	-14.83 (10.13)	-1.49 (-2.55, -0.43)	.006
24-h	-13.40 (9.42)	-14.30 (8.79)	-0.84 (-1.78, 0.11)	.08
Morning	-12.21 (13.92)	-15.52 (13.56)	-2.93 (-4.37, -1.49)	<.001

© 2025 Ye R et al. *JAMA Network Open.*

eTable 8. Differences in Changes Between the Morning and Bedtime Dosing Groups in BP Load

	Morning dosing (n=352)	Bedtime dosing (n=368)	Adjusted difference	P-value
**24-h BP load, mean (SD), %**				
SBP	-41.98 (27.04)	-45.27 (26.42)	-3.39 (-7.29, 0.52)	.09
DBP	-34.79 (25.46)	-36.64 (25.80)	-1.74 (-5.43, 1.95)	.35
**Daytime BP load, mean (SD), %**				
SBP	-42. 57 (28.30)	-43.75 (28.46)	-1.48 (-5.59, 2.64)	.48
DBP	-37.25 (27.83)	-37.39 (27.48)	-0.12 (-4.02, 3.76)	.95
**Nighttime BP load, mean (SD), %**				
SBP	-41.34 (33.57)	-49.52 (30.80)	-8.19 (-12.90, -3.49)	.001
DBP	-30.30 (30.61)	-35.85 (30.24)	-5.52 (-9.99, -1.05)	.02

© 2025 Ye R et al. *JAMA Network Open.*

eTable 9. The Treatment Response Rate for OBPM at Week 4, 8, and 12

	Morning dosing	Bedtime dosing	P-value
Week 4	228 (69.1) (n=330)	269 (75.4) (n=357)	.07
Week 8	257 (81.6) (n=315)	275 (83.6) (n=329)	.50
Week 12	239 (80.2) (n=298)	277 (89.6) (n=309)	.001

The treatment response rate was used as an index to evaluate the efficacy of antihypertensive drugs, and defined as: SBP <140mmHg or a decrease >20mmHg, and DBP <90mmHg or a decrease>10mmHg.

© 2025 Ye R et al. *JAMA Network Open.*

eTable 10. The Proportions of Patients Receiving Dosage Titration During Follow-Ups and Mean BP Values

	1 OA			1.5 OA			2 OA		
	Morning dosing	Bedtime dosing	P-value	Morning dosing	Bedtime dosing	P-value	Morning dosing	Bedtime dosing	P-value
4-week (n=687), % BP, mean (SD), mmHg	127 (38.5)	169 (47.3)	.02	203 (61.5)	188 (52.7)	.02	-	-	-
Office SBP	122.02 (11.79)	123.16 (12.11)	.42	140.11 (14.10)	139.26 (15.69)	.57	-	-	-
Office DBP	76.17 (7.66)	76.94 (8.28)	.41	86.45 (10.82)	86.27 (10.18)	.86	-	-	-
Daytime SBP	121.70 (10.54)	123.93 (10.47)	.07	141.60 (12.53)	143.17 (13.00)	.23	-	-	-
Daytime DBP	76.05 (7.02)	76.98 (6.68)	.25	86.79 (7.83)	88.27 (8.27)	.07	-	-	-
Nighttime SBP	107.48 (9.86)	108.47 (10.81)	.42	126.86 (15.11)	126.54 (13.91)	.83	-	-	-
Nighttime DBP	67.86 (7.09)	67.49 (6.08)	.63	78.26 (8.75)	79.32 (8.68)	.23	-	-	-
24-h SBP	117.31 (9.30)	119.01 (9.36)	.12	136.87 (12.03)	137.84 (12.08)	.43	-	-	-
24-h DBP	73.58 (6.42)	74.09 (5.76)	.47	84.14 (7.33)	85.45 (7.60)	.09	-	-	-
Titrated due to isolated nighttime BP elevation, %	-	-	-	33 (10)	45 (12.6)	.28	-	-	-
8-week (n=644), % BP, mean (SD), mmHg	116 (36.8%)	149 (45.3%)	.03	98 (31.1%)	98 (29.8%)	.72	101 (32.1%)	82 (24.9%)	.04
Office SBP	122.25 (11.36)	122.26 (10.91)	.99	129.87 (11.64)	127.61 (14.19)	.23	135.46 (14.14)	137.01 (16.03)	.49
Office DBP	75.53 (7.92)	74.01 (7.63)	.11	81.98 (9.47)	80.09 (8.30)	.14	81.81 (10.16)	83.97 (11.49)	.18

© 2025 Ye R et al. *JAMA Network Open.*

eTable 11. Adverse Events Reported by Participants and the Incidence of Nocturnal Hypotension During the Study

Adverse events (n, %)	Morning dosing (n=352)	Bedtime dosing (n=368)	P-value
Cough	2 (0.6)	1 (0.3)	.62
Edema limbs	2 (0.6)	0 (0.0)	.24
Dizziness	7 (2.0)	4 (1.1)	.38
Vomiting	0 (0.0)	1 (0.3)	>.99
Headache	2 (0.6)	1 (0.3)	.62
Fatigue	0 (0.0)	1 (0.3)	>.99
Hypotension	1 (0.3)	3 (0.8)	.62
Fracture	0 (0.0)	0 (0.0)	-
Palpitations	0 (0.0)	2 (0.5)	.50
Nausea	1 (0.3)	1 (0.3)	>.99
Erectile dysfunction	1 (0.3)	0 (0.0)	.49
Acute kidney injury	2 (0.6)	0 (0.0)	.24
Urine output decreased	0 (0.0%)	0 (0.0%)	-
Blurred vision	0 (0.0)	0 (0.0)	-
Glaucoma	0 (0.0)	0 (0.0)	-
Optic nerve disorder	0 (0.0)	0 (0.0)	-
Nighttime BP <100/60mmHg	58 (16.5)	75 (20.4)	.18
Nighttime BP <90/50mmHg	6 (1.7)	6 (1.6)	.94

© 2025 Ye R et al. *JAMA Network Open.*

eTable 12. Adverse Events and the Incidence of Nocturnal Hypotension Stratified by Age

Adverse events (n, %)	Age⩾65			Age<65		
	Morning dosing (n=95)	Bedtime dosing (n=79)	P-value	Morning dosing (n=257)	Bedtime dosing (n=289)	P-value
Cough	1 (1.1)	0 (0.0)	.55	1 (0.4)	1 (0.3)	.72
Edema limbs	1 (1.1)	0 (0.0)	.55	1 (0.4)	0 (0.0)	.47
Dizziness	2 (2.1)	1 (1.3)	.57	5 (1.9)	3 (1.0)	.30
Vomiting	0 (0.0)	0 (0.0)	-	0 (0.0)	1 (0.3)	.53
Headache	1 (1.1)	0 (0.0)	.55	1 (0.4)	1 (0.3)	.72
Fatigue	0 (0.0)	0 (0.0)	-	0 (0.0)	1 (0.3)	.53
Hypotension	0 (0.0)	0 (0.0)	-	1 (0.4)	3 (1.0)	.36
Fracture	0 (0.0)	0 (0.0)	-	0 (0.0)	0 (0.0)	-
Palpitations	0 (0.0)	0 (0.0)	-	0 (0.0)	2 (0.7)	.28
Nausea	0 (0.0)	0 (0.0)	-	1 (0.4)	1 (0.3)	.72
Erectile dysfunction	0 (0.0)	0 (0.0)	-	1 (0.4)	0 (0.0)	.47
Acute kidney injury	1 (1.1)	0 (0.0)	.55	1 (0.4)	0 (0.0)	.47
Urine output decreased						
Blurred vision	0 (0.0)	0 (0.0)	-	0 (0.0)	0 (0.0)	-
Glaucoma	0 (0.0)	0 (0.0)	-	0 (0.0)	0 (0.0)	-
Optic nerve disorder	0 (0.0)	0 (0.0)	-	0 (0.0)	0 (0.0)	-
Nighttime BP <100/60mmHg	17 (17.9)	21 (26.6)	0.17	41 (16.0)	54 (18.7)	.40
Nighttime BP <90/50mmHg	2 (2.1)	1 (1.3)	0.57	4 (1.6)	5 (1.7)	.57

© 2025 Ye R et al. *JAMA Network Open.*

eTable 13. Adverse Events and the Incidence of Nocturnal Hypotension Stratified by Comorbidities

Adverse events (n, %)	With DM			Without DM		
	Morning dosing (n=55)	Bedtime dosing (n=66)	P-value	Morning dosing (n=297)	Bedtime dosing (n=302)	P-value
Cough	1 (1.1)	0 (0.0)	-	2 (0.7)	1 (0.3)	.49
Edema limbs	1 (1.8)	0 (0.0)	.46	1 (0.3)	0 (0.0)	.50
Dizziness	0 (0.0)	0 (0.0)	-	7 (2.4)	4 (1.3)	.35
Vomiting	0 (0.0)	0 (0.0)	-	0 (0.0)	1 (0.3)	.50
Headache	1 (1.1)	0 (0.0)	-	2 (0.7)	1 (0.3)	.49
Fatigue	0 (0.0)	1 (1.5)	.55	0 (0.0)	0 (0.0)	-
Hypotension	0 (0.0)	0 (0.0)	-	1 (0.3)	3 (1.0)	.32
Fracture	0 (0.0)	0 (0.0)		0 (0.0)	0 (0.0)	-
Palpitations	0 (0.0)	0 (0.0)	-	0 (0.0)	2 (0.7)	.25
Nausea	0 (0.0)	0 (0.0)	-	1 (0.3)	1 (0.3)	.75
Erectile dysfunction	0 (0.0)	0 (0.0)	-	1 (0.3)	0 (0.0)	.50
Acute kidney injury	1 (1.8)	0 (0.0)	.46	1 (0.3)	0 (0.0)	.50
Urine output decreased						
Blurred vision	0 (0.0)	0 (0.0)	-	0 (0.0)	0 (0.0)	-
Glaucoma	0 (0.0)	0 (0.0)	-	0 (0.0)	0 (0.0)	-
Optic nerve disorder	0 (0.0)	0 (0.0)	-	0 (0.0)	0 (0.0)	-
Nighttime BP <100/60mmHg	5 (9.1)	12 (18.2)	.15	53 (17.8)	63 (20.9)	.35
Nighttime BP <90/50mmHg	2 (3.6)	0 (0.0)	.21	4 (1.3)	6 (2.0)	.39
	With CKD			Without CKD		
Adverse events (n, %)	Morning dosing (n=10)	Bedtime dosing (n=5)	P-value	Morning dosing (n=342)	Bedtime dosing (n=363)	P-value

© 2025 Ye R et al. *JAMA Network Open.*

Adverse events (n, %)	With DM			Without DM		
Cough	1 (1.1)	0 (0.0)	-	2 (0.6)	1 (0.3)	.48
Edema limbs	1 (10.0)	0 (0.0)	.67	1 (0.3)	0 (0.0)	.49
Dizziness	0 (0.0)	1 (20.0)	.33	7 (2.0)	3 (0.8)	.15
Vomiting	0 (0.0)	0 (0.0)	-	0 (0.0)	1 (0.3)	.52
Headache	1 (1.1)	0 (0.0)	-	2 (0.6)	1 (0.3)	.48
Fatigue	0 (0.0)	0 (0.0)	-	0 (0.0)	1 (0.3)	.52
Hypotension	0 (0.0)	0 (0.0)	-	1 (0.3)	3 (0.8)	.34
Fracture	0 (0.0)	0 (0.0)	-	0 (0.0)	0 (0.0)	-
Palpitations	0 (0.0)	0 (0.0)	-	0 (0.0)	2 (0.6)	.27
Nausea	1 (10.0)	0 (0.0)	.67	0 (0.0)	1 (0.3)	.52
Erectile dysfunction	0 (0.0)	0 (0.0)	-	1 (0.3)	0 (0.0)	.49
Acute kidney injury	1 (10.0)	0 (0.0)	.67	1 (0.3)	0 (0.0)	.49
Urine output decreased						
Blurred vision	0 (0.0)	0 (0.0)	-	0 (0.0)	0 (0.0)	-
Glaucoma	0 (0.0)	0 (0.0)	-	0 (0.0)	0 (0.0)	-
Optic nerve disorder	0 (0.0)	0 (0.0)	-	0 (0.0)	0 (0.0)	-
Nighttime BP <100/60mmHg	1 (10.0)	1 (20.0)	.57	57 (16.7)	74 (20.4)	.21
Nighttime BP <90/50mmHg	0 (0.0)	0 (0.0)	-	6 (1.8)	6 (1.7)	.92

© 2025 Ye R et al. *JAMA Network Open.*

eTable 14. The Number of Missing Data Imputed for the ITT Analysis

	Missing number		
	Morning dosing	Bedtime dosing	Overall
4-week			
Office BP	22	11	33
Daytime BP	22	11	33
Nighttime BP	22	11	33
24-h BP	22	11	33
Morning BP	22	11	33
8-week			
Office BP	37	39	76
12-week			
Office BP	54	59	113
Daytime BP	54	59	113
Nighttime BP	54	59	113
24-h BP	54	59	113
Morning BP	54	59	113

© 2025 Ye R et al. *JAMA Network Open.*

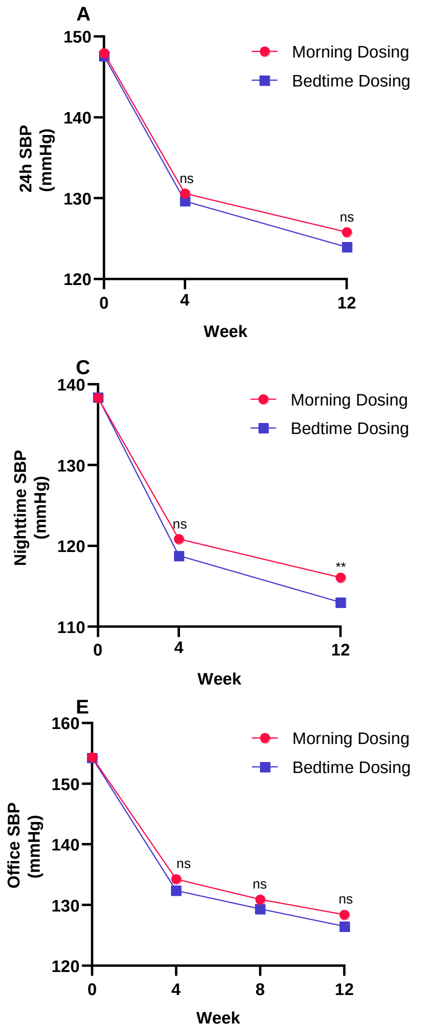

eFigure 1. The Mean SBP of Office and ABPM Values at Baseline and During Follow-Ups

*: P<0.05; **: P<0.01; ns: not significant

© 2025 Ye R et al. *JAMA Network Open.*

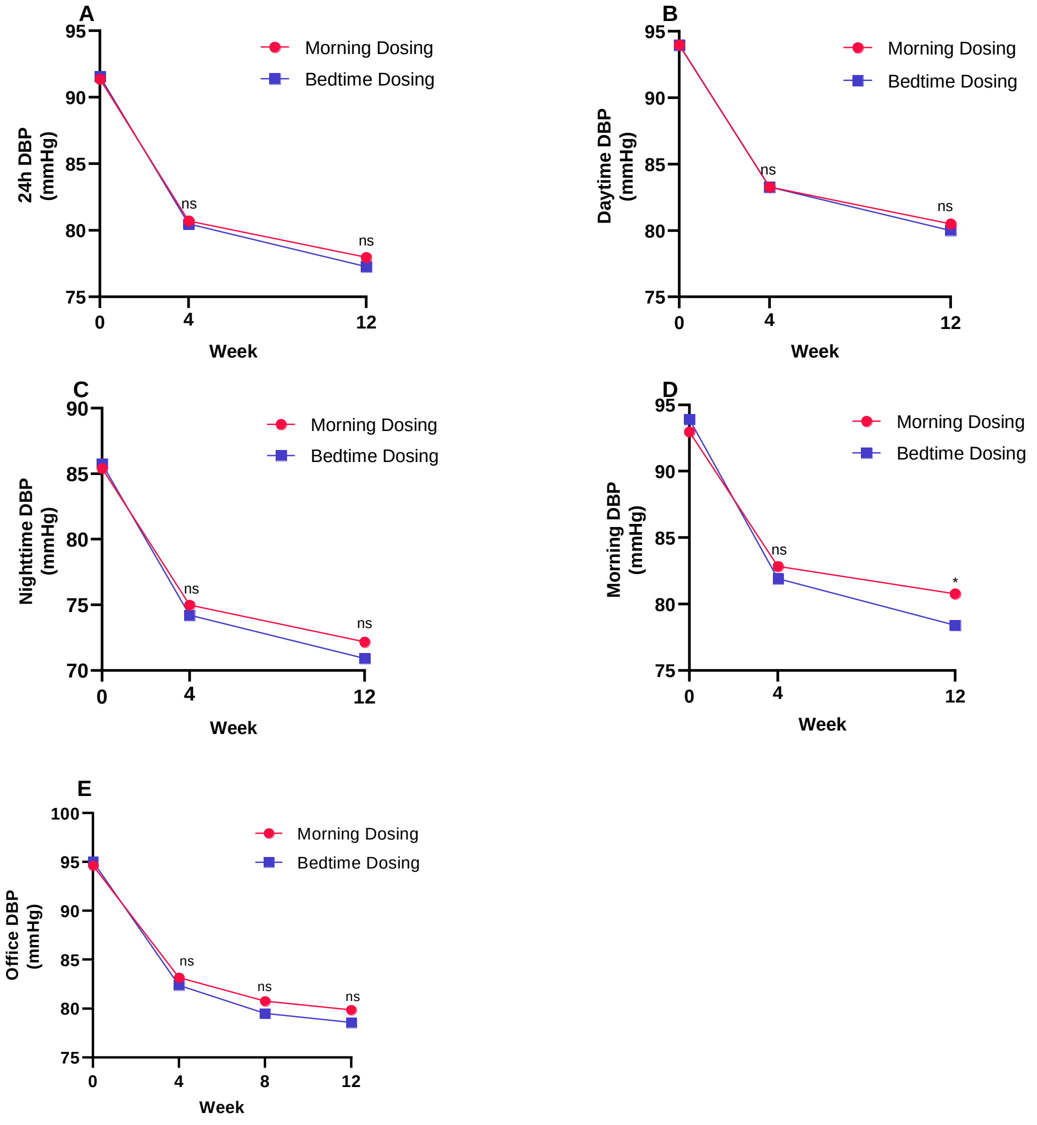

eFigure 2. The Mean DBP of Office and ABPM Values at Baseline and During Follow-Ups

*: P＜0.05; ns: not significant

© 2025 Ye R et al. *JAMA Network Open.*

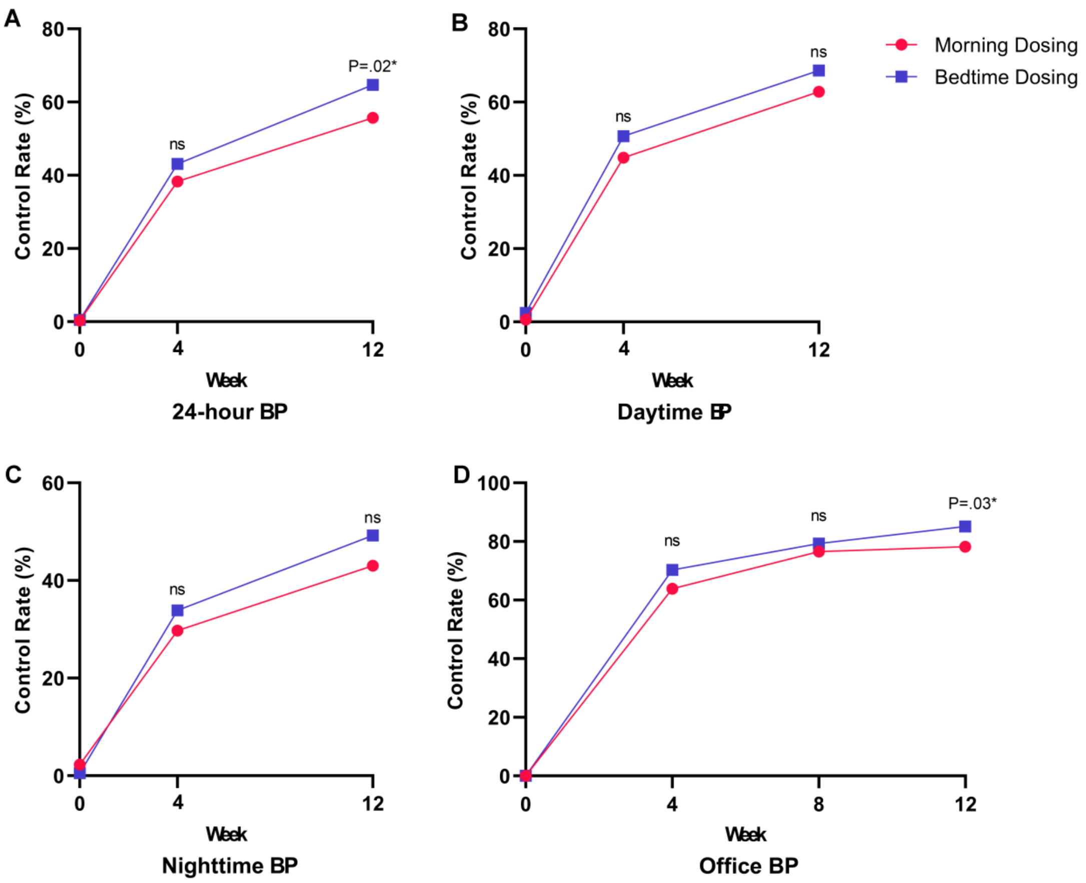

eFigure 3. Office and ABPM BP Control Rates at Baseline and During Follow-Ups

© 2025 Ye R et al. *JAMA Network Open.*

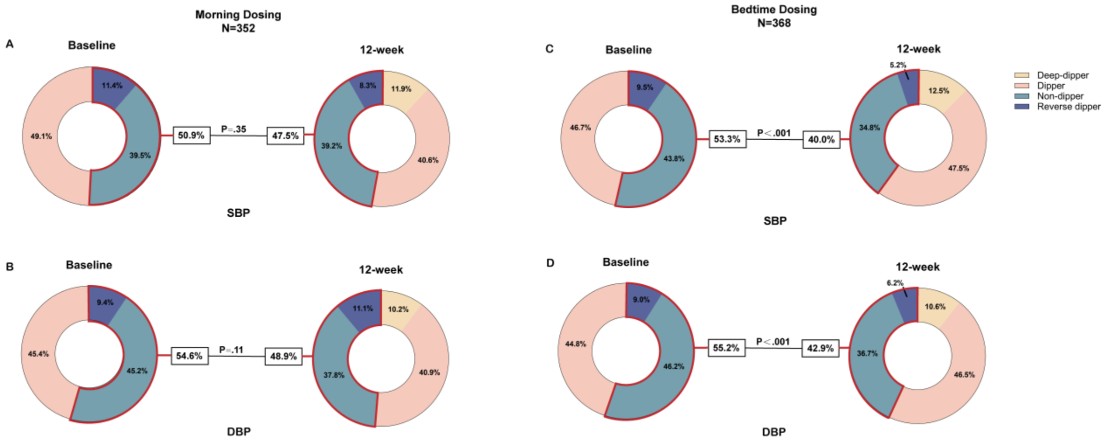

eFigure 4. The Distribution of Dipping Status for Morning Dosing and Bedtime Dosing Groups at Week 12**.** SBP: systolic blood pressure; DBP: diastolic blood pressure.

© 2025 Ye R et al. *JAMA Network Open.*

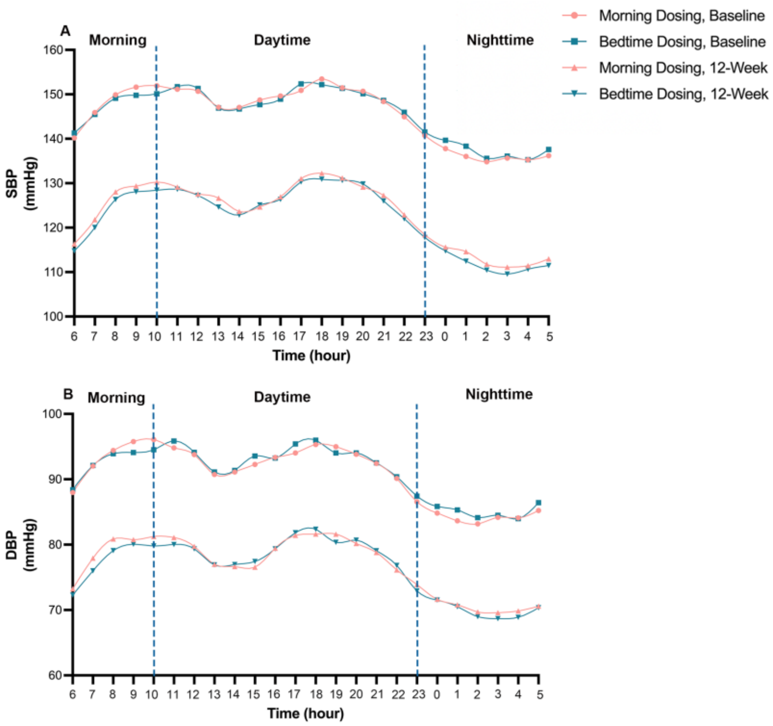

eFigure 5. Mean Hourly Ambulatory BP Profiles Illustrating the Overall BP Fluctuations Across All Patients at Baseline and Week 12 for SBP (A) and DBP (B)

© 2025 Ye R et al. *JAMA Network Open.*

Subgroup	Bedtime dosing	Morning dosing	Difference (95% CI)
**Age**			
<65	289	257	-2.03 (-4.40, 0.34)
⩾65	79	95	-6.18 (-10.51, -1.84)
**Sex**			
Male	203	206	-3.01 (-5.70, -0.33)
Female	165	146	-2.85 (-6.10, 0.40)
**Baseline office SBP**			
<155	193	169	-1.98 (-4.77, 0.82)
⩾155	175	183	-4.14 (-7.22, -1.05)
**BMI**			
<24	106	97	-2.34 (-6.26, 1.59)
⩾24	262	255	-3.21 (-5.66, -0.77)
**DM**			
without DM	302	297	-2.46 (-4.77, -0.15)
with DM	66	55	-6.21 (-10.86, -1.56)
**CKD**			
without CKD	363	342	-3.02 (-5.10, -0.93)
with CKD	5	10	1.48 (-18.70, 21.67)
**Drinking status**			
never/former	278	253	-3.21 (-5.64, -0.78)
current	90	99	-2.48 (-6.36, 1.41)
**Smoking status**			
never/former	298	267	-3.20 (-5.56, -0.84)
current	70	85	-2.51 (-6.90, 1.87)

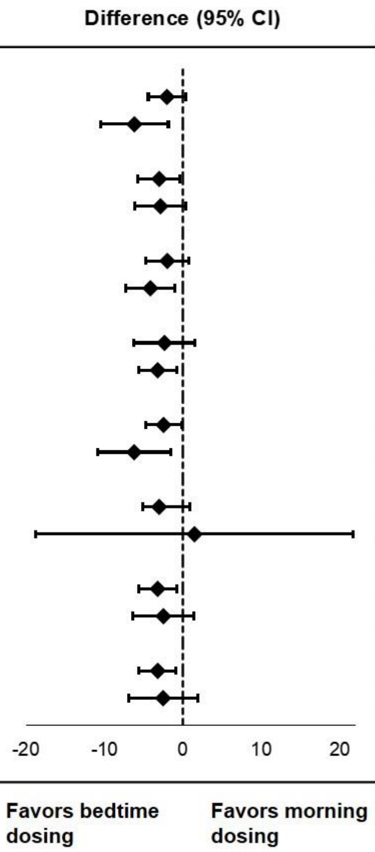

Figure 6. Differences in Nighttime SBP Between the Morning and Bedtime Dosing Groups at 12 Weeks in Patient Subgroups

© 2025 Ye R et al. *JAMA Network Open.*

eAppendix. Findings and Responsibilities of the Independent Monitoring Committee

The independent monitoring committee played a critical role in ensuring participant safety, data integrity, and ethical conduct throughout the trial. The responsibilities and findings of the independent monitoring committee are listed as follows:

1. Safety monitoring:

Responsibilities: Regularly reviewed safety data, including adverse events, serious adverse events, and laboratory results, to ensure participant safety. Provided recommendations to the investigators based on safety findings.

Findings: No safety concerns arose during the trial.

2. Interim analyses:

Responsibilities: Conducting interim analyses of efficacy and safety data to determine whether the trial should continue, be modified, or be stopped.

Findings: Recommended continuing the trial until the number of participants met the sample size calculation.

3. Ethical Oversight:

Responsibilities: Oversaw the ethical conduct of the trial, ensuring adherence to ethical principles, the study protocol, and the protection of participant rights.

Findings: (1) Two sites measured office blood pressure only once (instead of three times as required by the protocol) for the first recruited participant. This issue was corrected immediately. (2) Three sites did not adequately inform patients about the precautions for ambulatory blood pressure measurement. This issue was corrected immediately.

4. Data quality and integrity:

Responsibilities: Monitored data quality and integrity, identifying any inconsistencies or errors that could compromise the trial's validity.

Findings: Some sites failed to upload clinic data, such as fasting blood glucose and total

© 2025 Ye R et al. *JAMA Network Open.*

cholesterol. The feedback on missing data was delivered immediately to the physicians, nurses, or clinical research coordinators at each site.

5. Recruitment and retention monitoring:

Responsibilities: Monitored recruitment rates and participant retention.

Findings: For sites with slow recruitment rates or participants unwilling to attend the follow-ups, the committee provided strategies to improve recruitment and retention.

6. Reporting:

Responsibilities: Provided timely reports to the investigators and regulatory authorities.

Findings: Summarized and reported the above-mentioned issues every 2 weeks.

© 2025 Ye R et al. *JAMA Network Open.*
