# Supplementary material for: Morning vs Bedtime Dosing and Nocturnal Blood Pressure Reduction in Patients With Hypertension: The OMAN Randomized Clinical Trial
Source: JAMA Netw Open. 2025 Jul 9;8(7):e2519354. doi: 10.1001/jamanetworkopen.2025.19354 (PMC12242701; doi:10.1001/jamanetworkopen.2025.19354)
Supplement: Supplement 3. — Data Sharing Statement [file jamanetwopen-e2519354-s003.pdf]

Data Sharing Statement

Ye. Morning vs Bedtime Dosing and Nocturnal Blood Pressure Reduction in Hypertension. *JAMA Netw Open*. Published July 08, 2025. doi:10.1001/jamanetworkopen.2025.19354

Data
Additional Information: Chinese Clinical Trial Registry Identifier: ChiCTR2200059719, URL: https://www.chictr.org.cn/showproj.html?proj=169782.
Data available: Yes
Data types: Deidentified participant data
How to access data: How to access data: Xiaopingchen15@126.com
When available: With publication

Supporting Documents
Document types: None

Additional Information
Who can access the data: researchers whose proposed use of the data has been approved
Types of analyses: for a specified purpose
Mechanisms of data availability: with investigator support AND with a signed data access agreement
